# Angel or devil: the dual roles of 2,3,5,4′-tetrahydroxystilbene-2-O-β-D-glucopyranoside in the development of liver injury based on integrating pharmacological techniques: a systematic review

**DOI:** 10.3389/fphar.2025.1523713

**Published:** 2025-02-03

**Authors:** Jiajie Jiang, Qixiu Wang, Qiang Wu, Bobin Deng, Cui Guo, Jie Chen, Jinhao Zeng, Yaoguang Guo, Xiao Ma

**Affiliations:** ^1^ TCM Regulating Metabolic Diseases Key Laboratory of Sichuan Province, Hospital of Chengdu University of Traditional Chinese Medicine, Chengdu, China; ^2^ State Key Laboratory of Southwestern Chinese Medicine Resources, Chengdu University of Traditional Chinese Medicine, Chengdu, China; ^3^ Affiliated Hospital of Liaoning University of Traditional Chinese Medicine, Shenyang, China; ^4^ Chengdu Shuangliu Hospital of Traditional Chinese Medicine, Chengdu, China; ^5^ School of Pharmacy, Xian Medical University, Xi’an, China

**Keywords:** 2,3,5,4′-tetrahydroxystilbene-2-O-β-D-glucoside, hepatotoxicity, hepatoprotection, liver injury, systematic review

## Abstract

**Background and purpose:**

2,3,5,4′-tetrahydroxystilbene-2-O-β-D-glucoside (TSG) exhibits a dualistic pharmacological profile, acting as both a hepatoprotective and hepatotoxic agent, which is intricately linked to its interaction with multiple signaling pathways and its stereoisomeric forms, namely, cis-SG and trans-SG. The purpose of this study is to evaluate both the hepatoprotective and hepatotoxic effects of TSG and give therapeutic guidance.

**Methods:**

This study performed a systematic search of eight databases to identify preclinical literature up until March 2024. The CAMARADES system evaluated evidence quality and bias. STATA and Python were used for statistical analysis, including dose-effect maps, 3D maps and radar charts to show the dose-time-effect relationship of TSG on hepatoprotection and hepatotoxicity.

**Results:**

After a rigorous screening process, a total of 24 studies encompassing 564 rodents were selected for inclusion in this study. The findings revealed that TSG exhibited bidirectional effects on the levels of ALT and AST, while also regulating the levels of ALT, AST, TNF-α, IL-6, serum TG, serum TC, SOD, MDA, IFN-γ, and apoptosis rate. The histological analysis of liver tissue confirmed the regulatory effects of TSG, and a comprehensive analysis revealed the optimal protective dosage range was 27.27–38.81 mg/kg/d and the optimal toxic dosage range was 51.93–76.07 mg/kg/d. TSG exerts the dual effects on liver injury (LI) through the network of Keap1/Nrf2/HO-1/NQO1, NF-κB, PPAR, PI3K/Akt, JAK/STAT and TGF-β pathways.

**Conclusion:**

TSG could mediate the pathways of oxidation, inflammation, and metabolism to result in hepatoprotection (27.27–38.81 mg/kg/d) and hepatotoxicity (51.93–76.07 mg/kg/d).

## Graphical Abstract

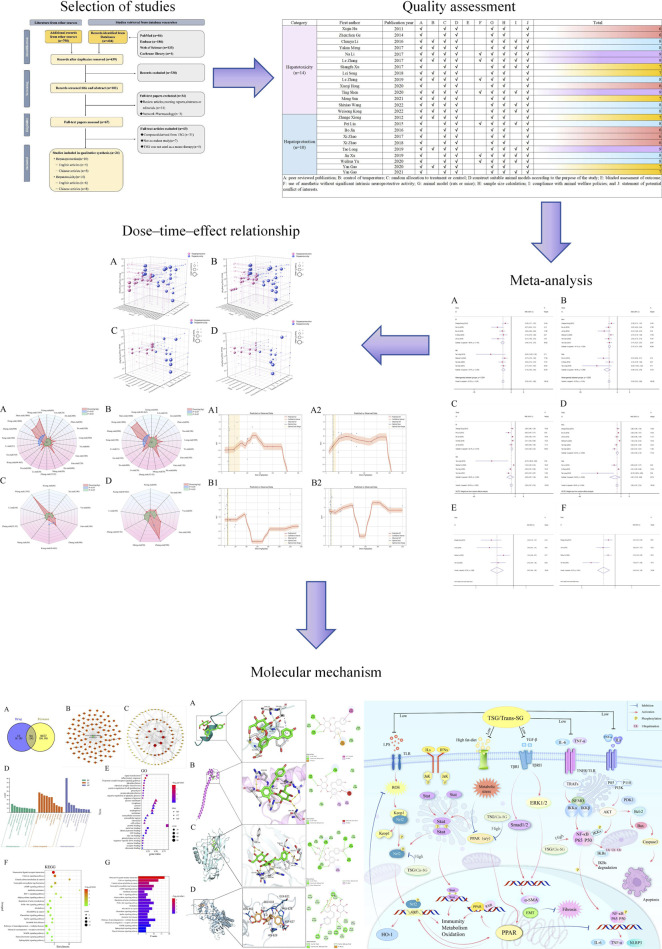

## 1 Introduction

The dynamic equilibrium between the toxic and therapeutic effects of pharmaceuticals used in the management of liver diseases presents a significant challenge that warrants meticulous examination. With the rising incidence of liver disease, it has become a significant public health concern, leading to 170,000 deaths each year in Europe (Acevedo, 2015). Liver injury (LI), often an early indicator of liver disease, can arise from various etiological factors, including alcohol consumption, infectious agents, immune dysregulation, and adverse drug reactions (Breit et al., 2023; Kirpich and McClain, 2012; Younossi et al., 2023). The pathological features of LI encompass inflammatory cell infiltration, steatosis, and ballooning degeneration of hepatocytes (Zhang et al., 2024b). Clinical presentations of LI encompass abnormal liver function test results, fever, nausea, vomiting, jaundice, and right upper quadrant pain (EASL et al., 2019; Knight, 2005). In the absence of timely intervention, LI may progress to liver failure, ultimately leading to mortality (Niewiński et al., 2020). Current standard therapeutic approaches for LI predominantly include antiviral medications, hepatoprotective strategies, and immunosuppressive agents such as corticosteroids, pioglitazone, and cholestyramine (Devarbhavi et al., 2023). However, these treatments can paradoxically induce drug-induced liver injury (DILI) due to their intrinsic hepatotoxic properties (Katarey and Verma, 2016). This highlights the urgent need to explore more effective and safer alternatives for the management of LI.

2,3,5,4′-tetrahydroxystilbene-2-O-β-D-glucoside (TSG; C20H22O9; MW = 406.38) is a bioactive compound extracted from the dried root of *Polygonum multiflorum* Thunb., which is a traditional herbal medicine and has garnered significant interest due to its complex nature regarding liver health (Lin et al., 2015a; Liu et al., 2022; Ma et al., 2015). TSG exhibits a dualistic pharmacological profile, acting as both a hepatoprotective and hepatotoxic agent, which is intricately linked to its interaction with multiple signaling pathways and its stereoisomeric forms, namely, cis-SG and trans-SG (Kong et al., 2022; Liu et al., 2022). The hepatoprotective effects of TSG are multifaceted, with its ability to activate the nuclear factor erythroid 2-related factor 2 (Nrf2)/heme oxygenase-1 (HO-1) pathway, a critical cellular defense mechanism against oxidative stress, being paramount (Gao et al., 2020; Yu W. et al., 2020). This activation bolsters the cell’s antioxidant capacity, thereby mitigating liver damage induced by reactive oxygen species (ROS) (Liu et al., 2022; Yu W. et al., 2020). Additionally, TSG is known to modulate the nuclear factor kappa-B(NF-κB) pathway to protect liver tissues, which interacts with phosphatidylinositol 3-kinase (PI3K)/protein kinase B (Akt) and Nrf2 pathways (Elbaset et al., 2024; Lawrence, 2009; Lin et al., 2015b). TSG could potentially counteract LI through the suppression of the NF-κB signaling cascade, which in turn stimulates the Nrf2–HO-1 signaling axis, and dampens the PI3K/Akt/NF-κB pathway (Gao et al., 2020; Lawrence, 2009; Lin et al., 2015b; Wang et al., 2020b; Xiong et al., 2012). However, TSG’s potential to cause liver damage has also been noted, particularly in relation to the peroxisome proliferator-activated receptor (PPAR) pathway, which has complex interactions with Janus kinase (JAK)/signal transducer and activator of transcription (STAT), Nrf2/HO-1, and NF-κB signaling pathways (Christofides et al., 2021; Zhang, 2017). TSG may inhibit Nrf2 activity by suppressing the PPAR/JAK/STAT/Nrf2 axis, while activating NF-κB, leading to LI (Meng et al., 2017; Shao et al., 2024; Zhang, 2017). Several studies suggested that TSG and its isomers, specifically the cis-form and trans-form, may exhibit differential effects on liver health. The cis-isomer, in particular, has been associated with an increased risk of LI, possibly through the inhibition of the peroxisome proliferator-activated receptor γ (PPARγ) pathway, which can exacerbate inflammation and immune responses (Kong et al., 2022; Meng et al., 2017). The trans-isomer, on the other hand, might have a more protective role under certain conditions, although the exact mechanisms are still under investigation (Liu et al., 2022). Furthermore, the duration and dosage of TSG medication are pivotal factors influencing its toxicity and therapeutic efficacy. However, there is a noticeable gap in the literature regarding the precise delineation of the toxic dose range for TSG. Despite the importance of understanding the safe dosage limits, current research has not yet provided a definitive framework for distinguishing the toxic dose thresholds of this medication.

It is crucial to recognize that the hepatoprotective and hepatotoxic effects of TSG may be interrelated and influenced by on various factors, including dosage, duration of exposure, and individual susceptibility. Further research is needed to fully elucidate the mechanisms by which TSG and its isomers influence liver health and to determine the safe therapeutic window for its use in treating liver diseases. Consequently, the objective of this study is to integrate pharmacological techniques to assess the influence of TSG in the development of LI and elucidate the dynamic processes through which TSG exerts its hepatoprotective and hepatotoxic effects.

## 2 Methods

### 2.1 Data sources and search strategy

**FIGURE 1:**
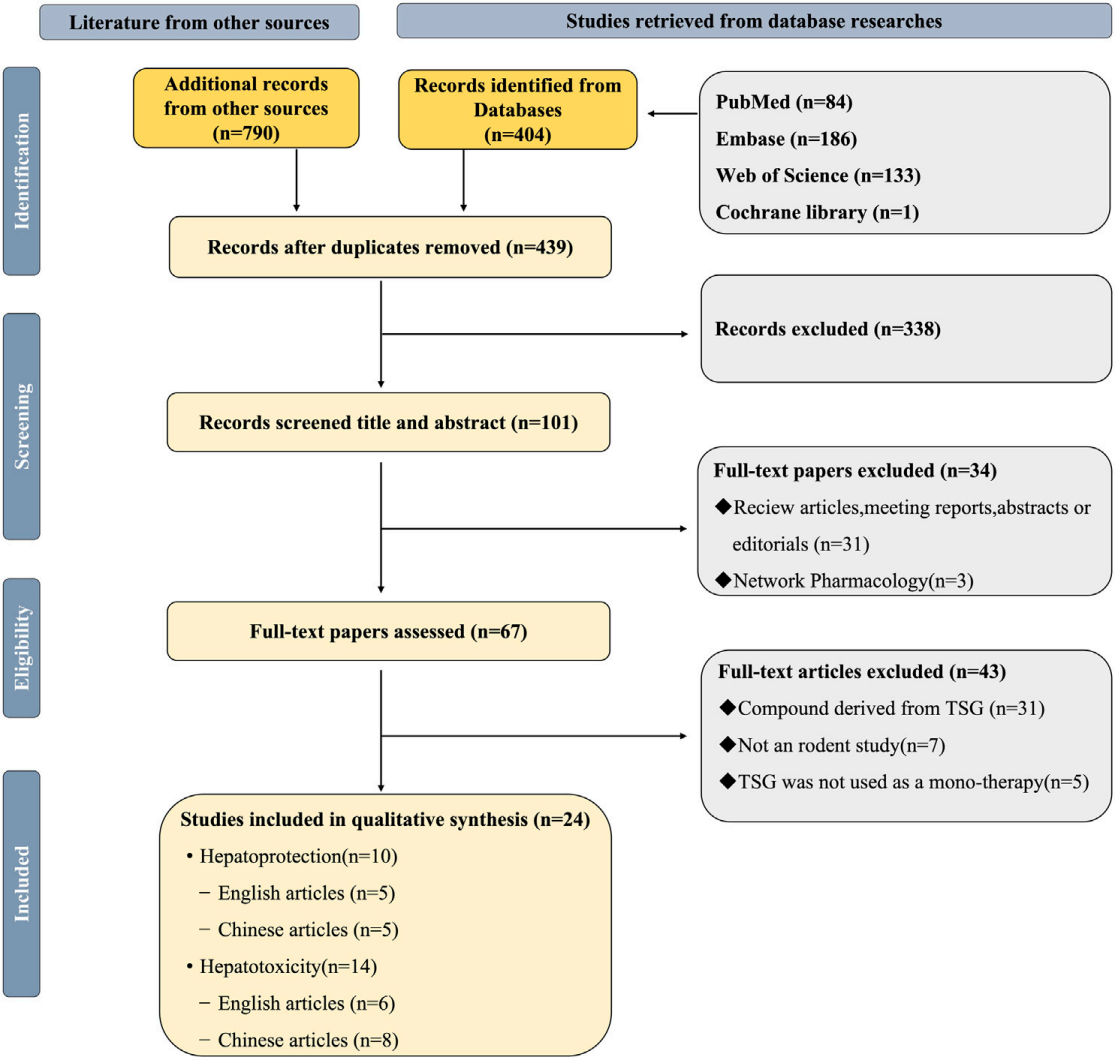
Selection of studies for this study.

This study accessed data from eight distinct repositories, which included four English-language databases and an equal number of Chinese-language databases: PubMed, Web of Science, the Cochrane Library, and Embase, alongside the China National Knowledge Infrastructure, Wanfang Medicine Online, the Chinese Science and Technology Journal Database, and the Chinese Biomedical Database (Ju et al., 2018; Liu et al., 2021; Luo et al., 2021; Xiong et al., 2019; Zheng et al., 2021). Up to March 2024, all eligible literatures were searched. The keywords were “2,3,5,4′-tetrahydroxystilbene-2-O-β-D-glucoside,” “liver injury,” “hepatoprotection,” and “hepatotoxicity.” (Figure 1; Supplementary Table 1).

### 2.2 Included criteria

#### 2.2.1 Studies on hepatoprotection

(1) Population: Studies must involve rats or mice. (2) Experimental design: At least one pair of intervention and control groups must be established, both consisting of liver injury models. (3) Intervention: The intervention groups should receive TSG monotherapy exclusively. (4) Control: Control groups must receive no treatment or a non-functional intervention. (5) Indicators: Alanine aminotransferase (ALT) and aspartate aminotransferase (AST) are essential experimental indicators. Tumor necrosis factor alpha (TNF-α), interleukin 6 (IL-6), serum triglyceride (TG), serum total cholesterol (TC), glutathione (GSH), malondialdehyde (MDA), and superoxide dismutase (SOD) are selective experimental indicators. (6) Quality evaluation: The quality assessment score must not be less than 5 points on the CAMARADES 10-point scale.

#### 2.2.2 Studies on hepatotoxicity

(1) Population: Studies must involve rats or mice. (2) Experimental design: At least one pair of intervention and control groups must be established. (3) Intervention: The intervention groups should receive TSG monotherapy exclusively. (4) Control: Control groups must receive no treatment or a non-functional intervention. (5) Indicators: ALT and AST are essential experimental indicators. TNF-α, IL-6, interferon gamma (IFN-γ), apoptosis rate, alkaline phosphatase (ALP), albumin (ALB), and total protein (TP) are selective experimental indicators. (6) Quality evaluation: The quality assessment score must be equal to or higher than 5 points on the CAMARADES 10-point scale.

### 2.3 Excluded criteria

(1) The animal subjects were not rats or mice. (2) No appropriate LI or normal animal models were selected for the study. (3) Lack of control group formation. (4) Intervention groups receiving interventions other than TSG monotherapy (e.g., Western medicine, traditional Chinese medicine, integrated medicine). (5) There are no necessary experimental indicators. (6) The quality evaluation result was less than 5 points.

### 2.4 Data extraction

Data extraction was performed by two independent researchers. The mean and standard deviation (SD) of continuous variables were estimated based on the collected experimental data using the Universal Desktop Ruler. The following information was extracted: (1) First author’s name and publication year; (2) Number, species (mice or rats), strain, sex, and weight of experimental animals; (3) Methodology for modeling and criteria for successful modeling; (4) Name, dosage, and frequency of drug administration; (5) Outcome indicators. Graphics were prioritized over digital text for reporting results (Table 1).

**TABLE 1:** The key characteristics of all 24 studies.

Author(s)/Year	Model category	Species	Gender (M/F)	Weight of the animal	Sample size (n)TSG/model	Drug dosage	Treatment courses	Main outcome indicators
Xiong 2012	NBI	Kunming mice	Male	18–22 g	8/8	TSG: Chinese liquor (56% vol), 12 mL/kg + TSG, 60 mg/kg Mod: Chinese liquor (56% vol), 12 mL/kg	6 days	ALT, AST, TNF-α, IL-6
LIn 2015	BI	Sprague Dawley rats	Male	347–461 g	7/7	TSG: High-fat diet + TSG, 24 mg/kg Mod: High-fat diet	12 weeks	ALT, AST, Serum TG Serum TC
Jin 2016	NBI	C57BL/6 mice	Male	18–22 g	15/15	TSG: 50% ethanol, 6 g/kg BW + TSG, 200 mg/kg Mod: 50% ethanol, 6 g/kg BW	3 days	ALT, AST, SOD
Zhao 2017	BI	Sprague Dawley rats	Male	180–220 g	7/7	TSG: Fat milk + TSG, 80 mg/kg Mod: Fat milk	6 weeks	GSH, MDA, Serum TG Serum TC, SOD
Zhao 2018	BI	Sprague Dawley rats	Male	160–200 g	7/7	TSG: High-fat emulsion + TSG, 80 mg/kg Mod: High-fat emulsion	6 weeks	ALT, AST, Serum TG Serum TC
Xu 2019	BI	C57BL/6 mice	Male	26–32 g	6/6	TSG: High-fat diet + TSG, 100 mg/kg Mod: High-fat diet	12 weeks	ALT, AST, GSH, IL-6 Serum TG, Serum TC TNF-α, MDA, SOD
Long 2019	NBI	Sprague Dawley rats	Female	200–250 g	3/3	TSG: CCl4 + TSG, 300 mg/kg Mod: CCl4	8 weeks	ALT, AST, GSH, MDA SOD
Yu 2020	NBI	C57BL/6 mice	Male	NM	10/10	TSG: Diethylnitrosamine, 100 mg/kg + TSG, 60 mg/kg Mod: Diethylnitrosamine, 100 mg/kg	5 days	ALT, AST, GSH, IL-6, TNF-α, MDA
Gao 2020	NBI	C57BL/6 mice	NM	NM	10/10	TSG: Acetaminophen, 350 mg/kg + TSG 180 mg/kg Mod: Acetaminophen, 350 mg/kg	3 days	ALT, AST, GSH, IL-6, TNF-α, SOD
Gao 2021	NBI	C57BL/6 mice	NM	20–30 g	15/15	TSG: Acetaminophen, 350 mg/kg + TSG, 120 mg/kg Mod: Acetaminophen, 350 mg/kg	7 days	ALT, AST, MDA, SOD
Hu 2011	N	Sprague Dawley rats	Male and Female	NM	10/10	TSG: Distilled water, 1 mL/100 g + TSG, 1,200 mg/kg Mod: Distilled water, 1 mL/100** **g	90 days	ALT, AST,TP
Ge 2014	N	ICR mice	Male and Female	18–22 g	10/10	TSG:Constant volume of 0.5% sodium carboxymethylcellulose + TSG, 185 mg/kg Mod: Constant volume of 0.5% sodium carboxymethylcellulose	10 days	ALT,AST
Meng 2017	LI	Sprague Dawley rats	Male	160–190 g	8/8/8/8 8/8	TSG 1. LPS, 2.8 mg/kg + Cis-SG, 50 mg/kg 2. LPS,2.8 mg/kg + Trans-SG, 50 mg/kg 3. Normal diet + Cis-SG, 50 mg/kg 4. Normal diet + Trans-SG, 50 mg/kg Mod 1. LPS, 2.8 mg/kg 2. Normal diet	3 days	ALT, AST, IL-6, TNF-α IFN-γ
Li 2017	LI	Sprague Dawley rats	Male	160–180 g	10/10 10/10	TSG 1. LPS, 2.8 mg/kg + Trans-SG, 31 mg/kg 2. Normal diet + Trans-SG, 31 mg/kg Mod 1. LPS, 2.8 mg/kg 2. Normal diet	5 days	ALT,AST
Zhang 2017	LI	Sprague Dawley rats	Male	180–200 g	8/8/8/8/8/8 8/8	TSG 1. LPS, 2.8 mg/kg + Cis-SG,7.56 mg/kg 2. LPS, 2.8 mg/kg + Cis-SG, 26.46 mg/kg 3. LPS, 2.8 mg/kg + Cis-SG, 52.92 mg/kg 4. Normal diet + Cis-SG, 7.56 mg/kg 5. Normal diet + Cis-SG, 26.46 mg/kg 6. Normal diet + Cis-SG, 52.92 mg/kg Mod 1. LPS, 2.8 mg/kg 2. Normal diet	10 h	ALT, AST, IL-6, TNF-α
Li 2017	LI	Sprague Dawley rats	Male	190–210 g	9/9/9/9 9/9	TSG 1. LPS, 2.8 mg/kg + Cis-SG, 30 mg/kg 2. LPS, 2.8 mg/kg + Trans-SG, 200 mg/kg 3. Normal saline + Cis-SG, 30 mg/kg 4. Normal saline + Trans-SG, 200 mg/kg Mod 1. LPS, 2.8 mg/kg 2. Normal saline	10 h	ALT, AST, IL-6, TNF-α IFN-γ
Xu 2017	LI	C57BL/6 mice	Male	18–22 g	10/10	TSG: Acetaminophen, 200 mg/kg + TSG, 400 mg/kg Mod: Acetaminophen, 200 mg/kg	12 h	ALT,AST
Song 2018	N	ICR mice	Male and Female	18–22 g	10/10	TSG: Constant volume of normal saline + TSG, 100 mg/kg Mod: Constant volume of normal saline	14 days	ALT,AST,ALP,ALB,TP
Zhang 2019	N	Sprague Dawley rats	Male	180–200 g	8/8	TSG: Normal saline + TSG, 500 mg/kg Mod: Normal saline	7 h	ALT, AST, IL-6, TNF-α IFN-γ
Hong 2020	N	Sprague Dawley rats	Male	150–180 g	6/6	TSG: Normal saline + TSG, 1,000 g/kg Mod: Normal saline	28 days	ALT,AST,ALP
Shen 2020	N	Sprague Dawley rats	Male and Female	80–100 g	10/10	TSG: Distilled water, 1 mL/100 g + TSG, 1,000 mg/kg Mod: Distilled water, 1 mL/100 g	90 days	ALT,AST,ALP
Sun 2021	N	C57BL/6 mice	NM	NM	6/6	TSG: Normal saline + TSG, 400 mg/kg Mod: Normal saline	15 days	ALP,TNF-α
Wang 2022	N	ICR mice	Male	18–20 g	6/6	TSG: Normal saline + TSG, 1,345 mg/kg Mod: Normal saline	28 days	ALT,ALP,TPALB
Kong 2022	LI	Balb/c mice	Female	NM	6/6/6/6 6	TSG 1. LPS, 0.5 mg/kg + Cis-SG, 0.18 mg/kg + Trans-SG, 4.8 mg/kg 2. LPS, 0.5 mg/kg + Cis-SG, 0.45 mg/kg + Trans-SG, 18 mg/kg 3. LPS, 0.5 mg/kg + Cis-SG, 0.45 mg/kg + Trans-SG, 18 mg/kg Mod: LPS, 0.5 mg/kg	14 days	ALT, AST, IL-6, TNF-a

Abbreviations: Green area represents the subject of TSG’s hepatoprotection (n = 8); Blue area represents the subject of TSG’s hepatotoxicity (n = 13). NBI, non-biomacromaolecule induced; BI, biomacromaolecule induced; Mod, model; N, normal; NM, not mentioned; LI, liver injury; ICR, institute of cancer research; ALT, alanine aminotransferase; AST, aspartate aminotransferase; SOD, superoxide dismutase; TNF-α, tumor necrosis factor alpha; MDA, malondialdehyde; GSH, glutathione; IL-6, interleukin 6; ALP, alkaline phosphatase; ALB, albumin; TP, total protein; IFN-γ, interferon gamma.

### 2.5 Risk of bias and quality of evidence

**FIGURE 2:**
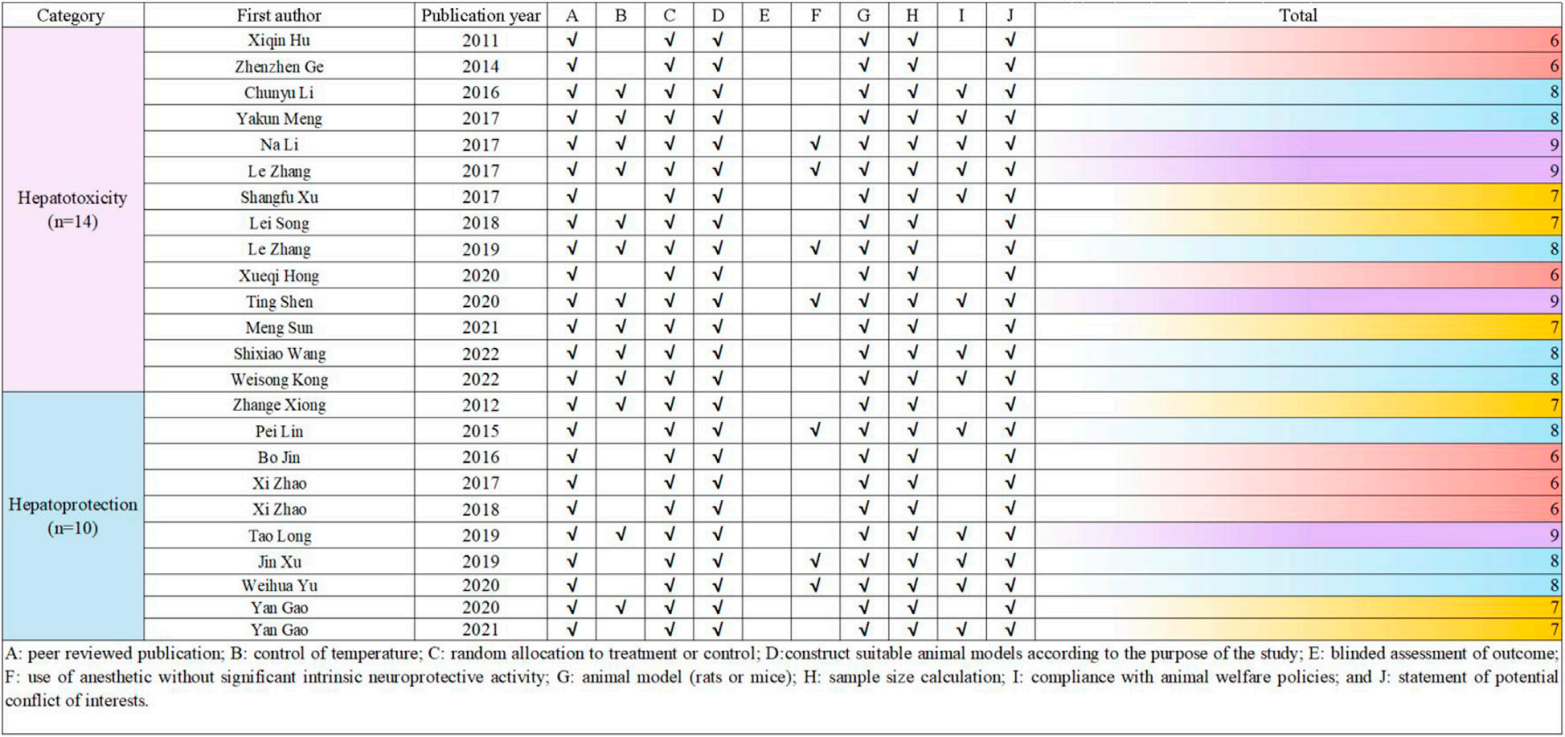
Risk of bias and quality assessment scores for included study.

The methodological quality of the included studies was independently assessed by two researchers using the CAMARADES (Collaborative Approach to Meta-Analysis and Review of Animal Data from Experimental Studies) 10-point scale (Macleod et al., 2004). Due to the specific nature of the study, the evaluation criteria were optimized by the researchers. In case of disagreements, the corresponding author acted as an arbitrator. The detailed method is presented in Figure 2.

### 2.6 Quantitative synthesis and statistical analyses

The study utilized STATA 16.0 software for conducting statistical analyses. Statistical significance was defined as p values less than 0.05 (p < 0.05). The results were assessed using the standardised mean difference (SMD) and the corresponding 95% confidence interval (95% CI). Heterogeneity between studies was evaluated using the I-squared (I^2^) test, with a random-effects model applied for I^2^ >50% and a fixed-effects model for I^2^ ≤ 50%. Results with an I^2^ of less than 50% were considered to have insignificant heterogeneity. Subgroup analysis was conducted for exploring whether the hepatoprotective effect of TSG would be affected by differences in species and modeling methods, including animal species subgroups (rats, mice) and modeling methods subgroups [non-biomacromolecule induced (NBI), biomacromolecule induced (BI)]. Additionally, subgroup analysis was performed for exploring whether the hepatotoxicity of TSG is related to species, modeling methods, and isomers, including animal models subgroups [normal(N) rodents, LI rodents], animal species subgroups (rats, mice), and isomers subgroups (cis-SG, trans-SG, as well as cis-SG and trans-SG). A sensitivity analysis and Egger’s test were carried out to ensure the credibility of the results for drawing inferences.

### 2.7 The dose-time-effect relationship and machine learning

In this study, the time unit for all included experiments was standardized to weeks (W). The dose-time-effect/toxicity relationship of TSG on the liver was visualized using 3D maps and radar charts. Four datasets with 81 diverse samples was collected to analyze the impact of intervention dosage on ALT and AST levels, a measure of TSG’s dual effects. The data underwent standardization using z-scores for consistency, enhancing model training efficiency and interpretability. A gradient boosting regression model was employed for precise prediction, with the data split into 8:2 training and test sets. The Radial Basis Function (RBF) kernel captured nonlinear relationships, and mean squared error (MSE) served as the metric for model evaluation, guiding dosage optimization to maximize ALT and AST level intervention. The LOWESS method was utilized for visualizing the relationship between dosage and variable effects, with confidence intervals plotted for clarity. Model performance was gauged by MSE, with lower values indicating better fit. Python (3.12.3) and Stata (16.0) were the analytical tools of choice. This study demonstrates a systematic approach to optimizing dosage through data-driven modeling and analysis.

### 2.8 Network pharmacology-based analysis

#### 2.8.1 Acquisition of TSG-related targets

Utilizing the SuperPred (https://prediction.charite.de/) and the BATMAN database (http://bionet.ncpsb.org.cn/batman-tcm/#/home), we conducted a comprehensive search to identify all potential targets of TSG. Subsequently, we refined the list of targets by aligning them with the UniProt database (https://www.uniprot.org/) to standardize the gene nomenclature. This process involved the exclusion of human-specific genes and the elimination of any invalid or redundant targets, ensuring a curated and standardized set of gene names.

#### 2.8.2 Acquisition of LI-related targets

To identify LI-related targets, we conducted searches in the GeneCards (https://www.genecards.org/) and OMIM (https://www.omim.org/) databases using the keyword “liver injury.” The resulting disease-associated targets were then compiled into a single Excel spreadsheet. We eliminated any duplicate genes and cross-referenced the list with the Uniprot database to refine and validate the gene information for the disease targets.

#### 2.8.3 Assembly of a shared PPI network for TSG and LI targets

A Venn diagram approach was employed to pinpoint the overlapping targets between TSG and LI. Subsequently, these shared targets were examined using the STRING database to gather data on protein-protein interactions (PPIs), with an emphasis on human proteins. The PPI network for the common targets was then graphically represented using Cytoscape 3.8.2, where the size and color of the nodes were adjusted to reflect their connectivity within the network.

#### 2.8.4 Go analysis and KEGG pathway enrichment analysis

The overlapping genes identified for TSG and LI were submitted to the DAVID database (https://david.ncifcrf.gov/summary.jsp) for comprehensive functional annotation. This resource is adept at evaluating the biological process (BP), cellular component (CC), and molecular function (MF) associated with the genes. The GO analysis elucidates the roles, pathways, and cellular contexts in which these genes are enriched. Additionally, the KEGG database (https://www.genome.jp/kegg/) serves as a repository for the systematic analysis of gene functions. The synthesis of GO and KEGG enrichment analyses facilitates a deeper understanding of the genes’ functional profiles and the potential pathways that link drugs to diseases. The visualization of the data was achieved by selecting the top 10 GO categories and the top 20 KEGG pathways based on the lowest P-values, which were then depicted using bar and bubble charts for a clear presentation.

### 2.9 Molecular docking

Two distinct databases served as repositories for the chemical compounds and molecular ligands: the PubChem database (https://pubchem.ncbi.nlm.nih.gov) and the RCSB Protein Data Bank (https://www.rcsb.org/structure). For the molecular docking procedures, AutoDockTools version 1.5.6 and AutoDock Vina version 4.2 were the chosen software tools. The detailed docking workflow is as follows.

1. The molecular framework of TSG was retrieved from the PubChem database and subsequently transformed into a three-dimensional configuration using ChemDraw, which also optimized the molecular energy. This 3D model was processed through AutoDockTools 1.5.6, and the output was stored in pdbqt format.
2. The ligands were sourced from the RCSB protein repository. After importing them into PyMOL, they underwent dehydration and hydrogenation processes, preparing them for subsequent separation into individual ligands. AutoDockTools 1.5.6 was then utilized to create a docking grid box centered on the active site of the target proteins, with the configuration saved in pdbqt format.
3. AutoDock Vina, specifically version 1.1.2, was deployed for docking the potential targets with the active compounds and for assessing the free binding energies.
4. For the visualization and analysis of molecular interactions, PyMOL version 2.6 and Discovery Studio 2019 were the software applications utilized.

## 3 Result

### 3.1 Comprehensive literature selection and study quality

A total of 1,184 articles were initially identified using specific keywords, comprising 404 articles from English databases and 790 articles from Chinese databases. After eliminating 745 duplicate articles, the researchers proceeded with evaluating the remaining 439 articles. Following a thorough review of titles and abstracts based on inclusion and exclusion criteria, 101 articles were excluded. Subsequently, 34 articles, including those related to TSG reviews, conference reports, abstracts, editorials, and web pharmacology, were further eliminated. Finally, after full-text reviews, 43 articles were excluded, resulting in a meta-analysis comprising 24 publications (Bo, 2016; Gao et al., 2020; Kong et al., 2022; Song et al., 2018; Li C. et al., 2017; Lin et al., 2015b; Long et al., 2019; Meng, 2021; Meng et al., 2017; Li N. et al., 2017; Shen et al., 2020; Wang et al., 2022; Xi, 2017; Xi, 2018; Hu et al., 2011; Xu et al., 2019; Xu et al., 2017; Xueqi, 2020; Gao, 2021; Yu W. et al., 2020; Zhang, 2017; Zhang et al., 2019; Xiong et al., 2012; Zhenzhen et al., 2014) (Figure 1).

To evaluate the quality of the research methodology, a revised CAMARADES checklist was applied, consisting of 10 distinct criteria. The inclusion criteria included publication in peer-reviewed journals, maintenance of appropriate temperature conditions, the use of relevant rodent models that matched the research goals, random assignment of subjects in the experiments, unbiased evaluation of outcomes, clear documentation of anesthesia protocols without significant inherent neuroprotective properties, calculation of sample sizes, compliance with ethical guidelines for animal research, and the revelation of any potential conflicts of interest.

Out of the 24 reviewed papers, each employed suitable rodent models with well-defined experimental groupings, along with thorough reporting on sample sizes and the declaration of potential conflicts of interest. However, only seven papers specifically addressed the use of anesthesia without neuroprotective effects, eleven papers omitted references to animal welfare guidelines, and none reported on blinded outcome assessments. The quality scores varied from 6 to 9, with six papers receiving a score of 6 (25.00%), another six scoring 7 (25.00%), eight papers scoring 8 (33.33%), and four papers achieving a score of 9 (16.67%). A graphical representation of the methodological quality for each study is depicted in Figure 2.

### 3.2 Basic information and features of the articles included

Sufficient information was available in the 24 papers to conduct a meta-analysis. These trials involved a total of 564 rodents, with 324 assigned to the treatment group and the remaining rodents serving as the control group (Table 1).

The animals’ weights in the studies ranged from 18 g to 250 g, categorized into five groups based on species distribution: Kunming mice (2.84%, 16/564), ICR mice (9.22%, 52/564), C57BL/6 mice (25.53%, 144/564), Balb/c mice (5.32%, 30/564), and Sprague Dawley Rats (57.09%, 322/564). Rats constituted 57.09% (322/564) of the total rodents, with mice comprising 42.91% (242/564).

Furthermore, all experiments on hepatoprotection were divided into rats (27.27%, 48/176) and mice (72.73%, 128/176) subgroups, with non-biomacromolecule-induced (NBI) (43.81%, 76/176) and biomacromolecule-induced (BI) (56.82%, 100/176) subgroups. Hepatotoxicity studies were categorized into rats (58.97%, 138/234) and mice (41.03%, 96/234) subgroups, as well as normal (N) (56.41%, 132/234) and liver injury (LI) (43.59%, 102/234) subgroups.

The daily TSG dosage ranged from 4.98 mg/kg to 1,345 mg/kg, administered for up to 90 days. For the two TSG isomers, 38 experiments involving 288 rodents examined the hepatotoxic effects of cis and trans isomers. Among the 288 mice, subgroups were based on animal modeling methods and TSG isomers: normal (N) (43.75%, 126/288) and liver injury (LI) (56.25%, 162/288); cis-SG (C) (56.94%, 164/288), trans-SG (T) (30.56%, 88/288), as well as cis-SG and trans-SG (C.T) (12.50%, 36/288) subgroups.

### 3.3 Protective effects of TSG on LI

The impact of TSG therapy on LI was evaluated by measuring the levels of ALT, AST, TNF-α, and IL-6, which were the primary outcomes. Additionally, the levels of GSH, MDA, SOD, serum TG and serum TC were also affected by TSG treatment (Supplementary Tables 2, 3). Histological analysis of 10 included articles of liver tissues from LI animals showed significant signs of inflammation, hepatocyte swelling, and hepatocellular necrosis (Bo, 2016; Gao et al., 2020; Lin et al., 2015b; Long et al., 2019; Xi, 2017; Xi, 2018; Xu et al., 2019; Gao, 2021; Yu W. et al., 2020; Xiong et al., 2012). Further analysis of this study to find out the optimal protective dosage range was 27.27–38.81 mg/kg/d.

#### 3.3.1 TSG improves the primary outcomes of LI

##### 3.3.1.1 ALT levels

**FIGURE 3:**
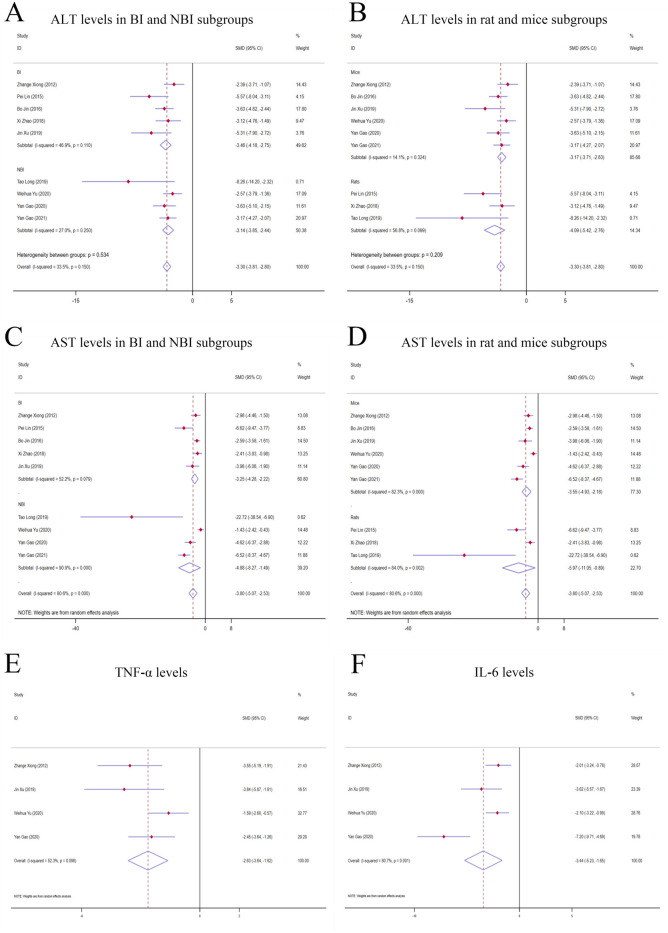
Forest plot (effect size and 95% CI) of TSG’s hepatoprotective roles on ALT, AST, TNF-α and IL-6. **(A)** ALT levels in BI and NBI subgroups; **(B)** ALT levels in rat and mice subgroups; **(C)** AST levels in BI and NBI subgroups; **(D)** AST levels in rat and mice subgroups; **(E)** TNF-α levels; **(F)** IL-6 levels. Abbreviations: 95% CI, 95% confidence interval; ALT, alanine aminotransferase; AST, aspartate aminotransferase; TNF-α, tumor necrosis factor alpha; IL-6, interleukin 6; BI, biomacromolecule induced; NBI, non-biomacromolecule induced.

Given the low degree of variability (I^2^ < 50%), a fixed-effects model was applied for the analysis. The findings indicated a substantial decrease in ALT levels for the TSG-intervention groups when contrasted with the LI model groups [n = 162, *95% CI* (-3.81,-2.80), *SMD* = |-3.30|, *I* ^*2*^ = 33.5%, *P-value* <0.0001] (Figures 3A, B; Supplementary Table 2).

##### 3.3.1.2 AST levels

Due to considerable variability among the studies (I^2^ > 50%), a random-effects model was implemented for the analysis. The findings demonstrated a noteworthy divergence in the levels of AST between the TSG and LI model groups, favoring the TSG groups with lower AST levels [n = 162, *95% CI* (-5.07,-2.53), *SMD* = |-3.80|, *I* ^*2*^ = 80.6%, *P-value* <0.0001] (Figures 3C, D; Supplementary Table 2).

##### 3.3.1.3 TNF-α levels

A considerable degree of heterogeneity (I^2^ > 50%) was observed, prompting the use of a random-effects model for the analysis. The data analysis revealed that the TSG group exhibited significantly lower TNF-α levels compared to the LI model groups [n = 68, *95% CI* (-3.64, −1.62), *SMD* = |-2.63|, *I* ^*2*^ = 52.3%, *P-value* <0.0001] (Figure 3E; Supplementary Table 2).

##### 3.3.1.4 IL-6 levels

Random-effects analyses showed variations in IL-6 levels among the rodent models in the study. The IL-6 levels in the TSG groups were notably lower than those in the control groups [n = 68, *95% CI* (-5.23,-1.65), *SMD* = |-3.44|, *I* ^*2*^ = 80.7%, *P-value* <0.0001] (Figure 3F; Supplementary Table 2).

#### 3.3.2 TSG effects on secondary outcomes of LI

##### 3.3.2.1 GSH levels

The random-effects model analysis indicated pronounced disparities in the levels of GSH between the TSG and LI model groups. It was found that the TSG groups had considerably elevated GSH levels in contrast to the LI model groups [n = 75, *95% CI* (1.97,5.26), *SMD* = |3.61|, *I* ^*2*^ = 75.1%, *P-value* <0.0001], as depicted in Supplementary Figure 1A; Supplementary Table 2.

##### 3.3.2.2 MDA levels

The random-effects model analysis exposed significant variations in the levels of MDA between the TSG and LI model groups. The TSG groups displayed substantially reduced MDA levels relative to the LI model groups [n = 82, *95% CI* (-3.89,-1.44), *SMD* = |-2.66|, *I* ^*2*^ = 66.7%, *P-value* <0.0001], as illustrated in Supplementary Figure 1B; Supplementary Table 2.

##### 3.3.2.3 SOD levels

Given the considerable heterogeneity (I^2^ > 50%), a random-effects model was utilized for a more in-depth analysis. The results demonstrated a marked difference in SOD levels between the TSG and LI model groups, with the TSG groups showing an increase in SOD activity [n = 112, *95% CI* (1.64,4.09), *SMD* = |2.87|, *I* ^*2*^ = 74.6%, *P-value* <0.0001], which is detailed in Supplementary Figure 1C; Supplementary Table 2.

##### 3.3.2.4 Serum TG levels

Based on the random-effects analysis, there were observed differences in serum TG levels among the animal models in the study. The TSG groups exhibited lower serum TG levels in comparison to the LI groups [n = 54, *95% CI* (-7.15,-1.13), *SMD* = |-4.14|, *I* ^*2*^ = 86.6%, *P-value* = 0.007], as represented in Supplementary Figure 1D; Supplementary Table 2.

##### 3.3.2.5 Serum TC levels

The random-effects model analysis indicated significant variations in serum TC levels between the TSG and LI model groups. The TSG groups had significantly decreased serum TC levels compared to the LI model groups [n = 54, *95% CI* (-9.19,-1.61), *SMD* = |-5.40|, *I* ^*2*^ = 87.0%, *P-value* = 0.005], as depicted in Supplementary Figure 1E; Supplementary Table 2.

#### 3.3.3 Subgroup analysis of hepatoprotection studies

##### 3.3.3.1 Analysis of ALT levels in distinct subgroups

When analyzing the liver enzyme levels, it was found that the TSG groups had markedly lower levels of ALT compared to the LI model groups. The most notable decrease in ALT levels was observed in the BI subgroups [n = 86, 95% *CI* (-4.18, −2.75), *SMD* = |-3.46|, *I* ^*2*^ = 46.9%, *P-value* <0.0001], surpassing the reduction observed in the NBI subgroup [n = 76, 95% *CI* (-3.85,-2.44), *SMD* = |-3.14|, *I* ^*2*^ = 27.0%, *P-value* <0.0001] (Figure 3A; Supplementary Table 3). The TSG intervention proved to be effective in both mice [n = 128, 95% *CI* (-3.71,-2.63), *SMD* = |-3.17|, *I* ^*2*^ = 14.1%, *P-value* <0.0001] and rats [n = 34, 95% *CI* (-5.42,-2.76), *SMD* = |-4.09|, *I* ^*2*^ = 56.8%, *P-value* <0.0001] subgroups, with a more pronounced reduction in the latter (Figure 3B; Supplementary Table 3). The variability in results was primarily attributed to the rats and BI subgroups. The TSG intervention consistently reduced ALT levels across all analyzed subgroups, without any significant differences in its effectiveness.

##### 3.3.3.2 Analysis of AST levels in distinct subgroups

In the evaluation of AST levels, the TSG groups also demonstrated a significant reduction compared to the LI model groups. The TSG intervention was particularly effective in reducing AST levels in mice [n = 128, 95% *CI* (-4.93,-2.18), *SMD* = |-3.55|, *I* ^*2*^ = 82.3%, *P-value* <0.0001] and rats [n = 34, 95% *CI* (-11.05,-0.89), *SMD* = |-5.97|, *I* ^*2*^ = 84.0%, *P-value* = 0.021] subgroups (Figure 3C; Supplementary Table 3). The BI subgroup [n = 86, 95% *CI* (-4.25,-2.22), *SMD* = |-3.25|, *I* ^*2*^ = 52.2%, *P-value* <0.0001] and the NBI subgroup [n = 76, 95% *CI* (-8.27,-1.49), *I* ^*2*^ = 90.9%, *SMD* = |-4.88|, *P-value* = 0.005] both responded positively to TSG, with the NBI and rats subgroups showing a more significant reduction in AST levels (Figure 3D; Supplementary Table 3). TSG demonstrated greater efficacy in lowering AST levels within the NBI and rat subgroups. Heterogeneity was more pronounced in the mice, rat, and NBI subgroups, contrasting with the comparatively lower heterogeneity observed in the BI subgroups.

### 3.4 Hepatotoxic effects of TSG

The hepatotoxic potential of TSG was assessed by examining four critical biomarkers in a comprehensive review of 14 research studies: ALT, AST, TNF-α, and IL-6. In contrast to the N groups, where no substantial changes were noted, a marked elevation in these biomarkers was observed in the LI groups. The findings suggest that TSG may intensify liver damage, particularly influencing the levels of ALT and AST in the LI groups. The histological examination of 9 included articles showed significant hepatotoxic effects in liver tissue, including inflammatory cell infiltration, cell edema, and vacuolar cytoplasmic degeneration (Kong et al., 2022; Li C. et al., 2017; Meng, 2021; Meng et al., 2017; Wang et al., 2022; Xu et al., 2017; Xueqi, 2020; Zhang, 2017; Zhang et al., 2019). Further analysis of this study to find out the optimal toxic dosage range was 51.93–76.07 mg/kg/d (Supplementary Tables 4, 5).

#### 3.4.1 Primary indicators of TSG’s hepatotoxic effects

##### 3.4.1.1 ALT levels

**FIGURE 4:**
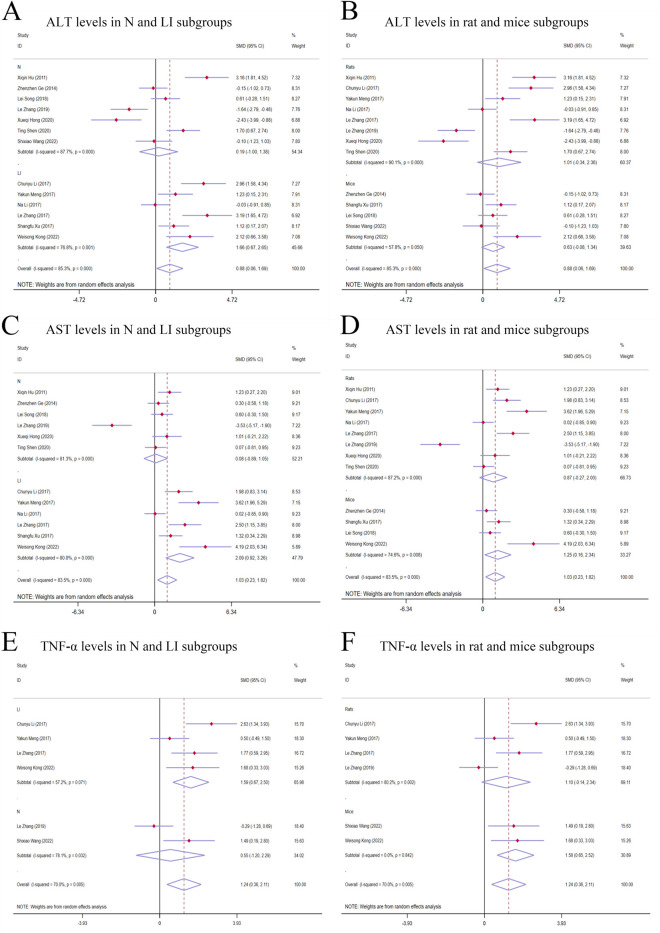
Forest plot (effect size and 95% CI) of TSG’s hepatotoxic roles on ALT, AST and TNF-α. **(A)** ALT levels in N and LI subgroups; **(B)** ALT levels in rat and mice subgroups; **(C)** AST levels in N and LI subgroups; **(D)** AST levels in rat and mice subgroups; **(E)** TNF-α levels in N and LI subgroups; **(F)** TNF-α levels in rat and mice subgroups. Abbreviations: 95% CI, 95% confidence interval; ALT, alanine aminotransferase; AST, aspartate aminotransferase; TNF-α, tumor necrosis factor alpha; N, normal; LI, liver injury.

Given the significant variability across studies (I^2^ > 50%), a random-effects model was applied for the statistical analysis. The findings indicated that the treatment of TSG resulted in a significant increase in ALT levels when compared with the control groups [n = 222, *95% CI* (0.06,1.69), *SMD* = |0.88|, *I* ^*2*^ = 85.3%, *P-value =* 0.034] (Figures 4A, B).

##### 3.4.1.2 AST levels

The random-effects model analysis highlighted a significant difference in AST levels between the groups treated with TSG and those in the control groups. The data suggested that TSG was linked to an increase in AST levels [n = 210, 95*% CI* (0.23,1.82), *SMD* = |1.03|, *I* ^*2*^ = 83.5%, *P-value* = 0.011] (Figures 4C, D).

##### 3.4.1.3 TNF-α levels

The presence of considerable heterogeneity (I^2^ > 50%) in the study data led to the application of a random-effects model. The analysis demonstrated that TSG-treated groups exhibited increased TNF-α levels compared to the control groups [n = 90, *95% CI* (0.36,2.11), *SMD* = |1.24|, *I* ^*2*^ = 70.0%, *P-value* = 0.006] (Figures 4E, F).

##### 3.4.1.4 IL-6 levels

**FIGURE 5:**
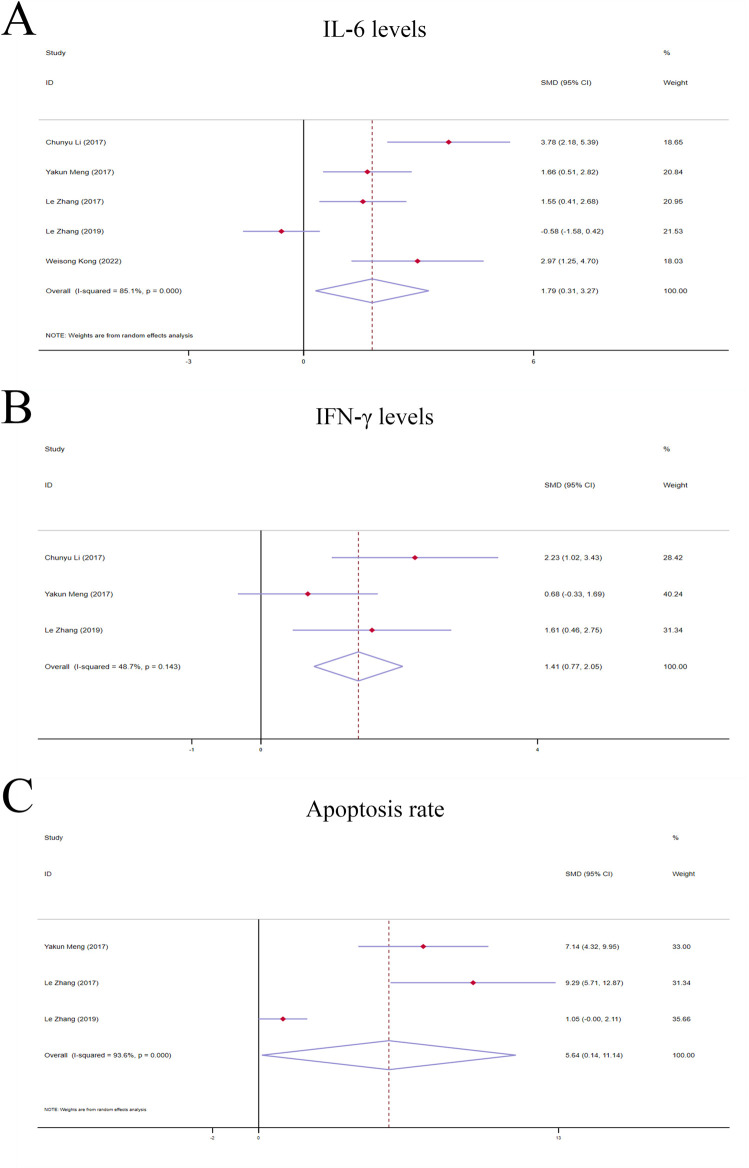
Forest plot (effect size and 95% CI) of TSG’s hepatoprotective roles on IL-6, IFN-γ and apoptosis rate. **(A)** IL-6 levels; **(B)** IFN-γ levels; **(C)** Apoptosis rate. Abbreviations: 95% CI, 95% confidence interval; IL-6, interleukin 6; IFN-γ, interferon gamma.

The random-effects model analyses revealed a notable variation in IL-6 levels among the rodent models under investigation. The IL-6 levels in the TSG groups were found to be markedly higher compared to the control groups [n = 78, *95% CI* (0.31,3.27), *SMD* = |1.79|, *I* ^*2*^ = 85.1%, *P-value* = 0.018] (Figure 5A).

#### 3.4.2 Secondary indicators of TSG’s hepatotoxic effects

##### 3.4.2.1 IFN-γ levels

A fixed-effects model analysis revealed significant variations in IFN-γ levels between the TSG groups and the control groups. The TSG groups demonstrated notably elevated the levels of IFN-γ relative to the control groups [n = 50, *95% CI* (0.77,2.05), *SMD* = |1.41|, *I* ^*2*^ = 48.7%, *P-value* <0.0001] (Figure 5B).

##### 3.4.2.2 Apoptosis rate

Due to the substantial heterogeneity observed (I^2^ > 50%), a random-effects model was utilized for the analysis. The findings showed that the TSG groups experienced a significantly increased rate of apoptotic cell death when compared to the control groups [n = 48, *95% CI* (0.14,11.14), *SMD* = |5.64|, *I* ^*2*^ = 93.6%, *P-value* = 0.044] (Figure 5C).

#### 3.4.3 Subgroup analysis of studies on hepatotoxicity

##### 3.4.3.1 Subgroup analysis of ALT levels

The subgroup analysis showed that TSG notably increased ALT levels in the LI subgroups [n = 102, *95% CI* (0.67,2.65), *SMD* = |1.66|, *I* ^*2*^ = 76.8%, *P-value =* 0.001], whereas no significant changes were detected in the N subgroups [n = 120, *95% CI* (-1.00,1.38), *SMD* = |0.19|, *I* ^*2*^ = 87.7%, *P-value =* 0.755] (Figure 4A). Toxic effects of TSG were observed in both mice [n = 84, 95% CI (-0.08, 1.34), SMD = |0.63|, *I* ^*2*^ = 57.8%, *P-value* = 0.083] and rats [n = 138, 95% CI (-0.34, 2.36), SMD = |1.01|, *I* ^*2*^ = 90.1%, *P-value* = 0.144] subgroups, with a more pronounced increase in ALT levels in the rats subgroups compared to the mice subgroups (Figure 4B). Rats models and N models were identified as the main sources of increased heterogeneity in the subgroup analysis.

##### 3.4.3.2 Subgroup analysis of AST levels

In the subgroup analysis based on modeling methods, AST levels were found to be elevated in the LI subgroups in response to TSG [n = 102, *95% CI* (0.92,3.26), *SMD* = |2.09|, *I* ^*2*^ = 80.8%, *P-value* < 0.001], whereas no significant differences were observed between the N subgroups and the control groups [n = 108, *95% CI* (-0.89,1.05), *SMD* = |0.08|, *I* ^*2*^ = 81.3%, *P-value =* 0.867] (Figure 4C). Both the N and LI subgroups contributed to the increased heterogeneity. Additionally, a trend of increasing AST levels was observed in the mice subgroups [n = 72, *95% CI* (0.16,2.34), *SMD* = |1.25|, *I* ^*2*^ = 74.6%, *P-value =* 0.025], whereas the rats subgroups did not exhibit statistically significant changes [n = 138, *95% CI* (-0.27,2.00), *SMD* = |0.87|, *I* ^*2*^ = 87.2%, *P-value =* 0.142] (Figure 4D).

##### 3.4.3.3 Subgroup analysis of TNF-α levels

The subgroup analysis based on modeling methods indicated a significant increase in TNF-α levels in the LI subgroups due to TSG [n = 62, *95% CI* (0.67,2.50), *SMD* = |1.59|, *I* ^*2*^ = 57.2%, *P-value =* 0.001], while no significant differences were noted in the N subgroups when compared to the control groups [n = 28, *95% CI* (-1.20,2.29), *SMD* = |0.55|, *I* ^*2*^ = 78.1%, *P-value =* 0.541] (Figure 4E). An increase in TNF-α levels was observed in both mice [n = 24, *95% CI* (0.65,2.52), *SMD* = |1.58|, *I* ^*2*^ = 0.0%, *P-value =* 0.001] and rats [n = 66, *95% CI* (-0.14,2.34), *SMD* = |1.10|, *I* ^*2*^ = 80.2%, *P-value =* 0.082] (Figure 4F). Rats models and N models were identified as the primary sources of increased heterogeneity in the subgroup analysis.

### 3.5 Analysis of the hepatotoxic effects of cis-SG and trans-SG

Cis-SG and trans-SG were two isomers of TSG. This study encompassed 38 experiments involving 288 rodents to explore the hepatotoxic effects of these two isomers. ALT, AST, TNF-α, and IL-6 levels were evaluated as primary indicators to assess the toxic effects of cis-SG and trans-SG. The levels of these indicators were elevated in the LI subgroups, C subgroups, and C.T subgroups, while there was no significant difference between T subgroups and N subgroup (Supplementary Tables 6, 7).

#### 3.5.1 The primary indicators of the hepatotoxic effects of cis-SG and trans-SG

##### 3.5.1.1 ALT levels

**FIGURE 6:**
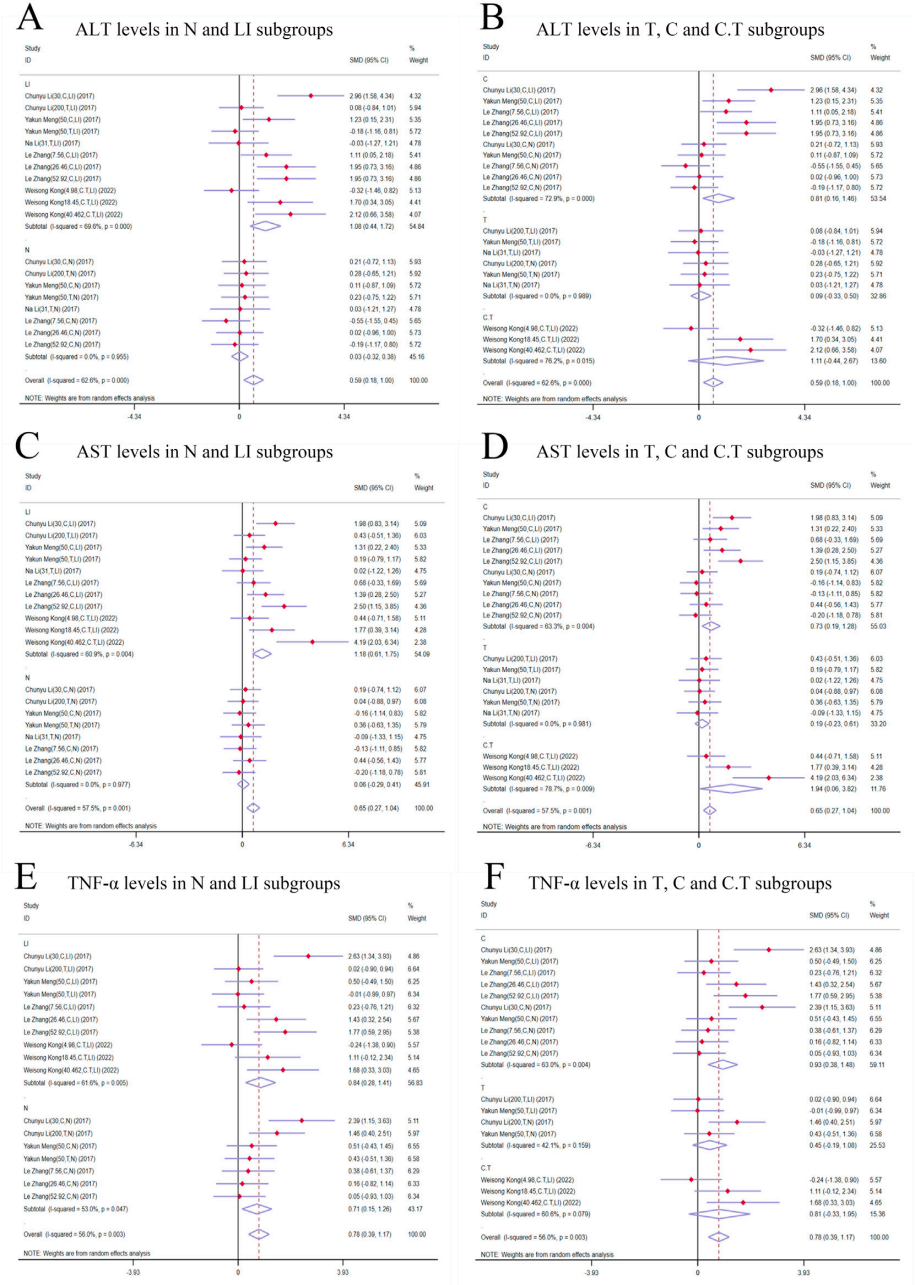
Forest plot (effect size and 95% CI) of cis/trans-SG’s hepatotoxic roles on ALT, AST and TNF-α. **(A)** ALT levels in N and LI subgroups; **(B)** ALT levels in T, C and C.T subgroups; **(C)** AST levels in N and LI subgroups; **(D)** AST levels in T, C and C.T subgroups; **(E)** TNF-α levels in N and LI subgroups; **(F)** TNF-α levels in T, C and C.T subgroups. Abbreviations: 95% CI, 95% confidence interval; ALT, alanine aminotransferase; AST, aspartate aminotransferase; TNF-α, tumor necrosis factor alpha; N, normal; LI, liver injury; T subgroup, trans-SG subgourp; C subgroup, cis-SG subgroup; C.T subgroup, cis-SG and trans-SG subgroup.

In terms of modeling methods subgroups, ALT levels significantly increased in the LI subgroups [n = 162, *95% CI* (0.44,1.72), *SMD* = |1.08|, *I* ^*2*^ = 69.6%, *P-value =* 0.001], while there was no difference in the N subgroups [n = 126, *95% CI* (-0.32,0.38), *SMD* = |0.03|, *I* ^*2*^ = 0.0%, *P-value =* 0.875] (Figure 6A). Both C and C.T subgroups showed toxic effects of TSG [C subgroups: n = 164, 95% *CI* (0.16,1.46), *SMD* = |0.81|, *I* ^*2*^ = 72.9%, *P-value* = 0.015; C.T subgroups: n = 36, 95% *CI* (-0.44,2.67), *SMD* = |1.11|, *I* ^*2*^ = 76.2%, *P-value* = 0.161], but no difference was observed in the T subgroups between trans-SG therapy groups and control groups [n = 88, 95% *CI* (-0.33,0.50), *SMD* = |0.09|, *I* ^*2*^ = 0.0%, *P-value* = 0.689] (Figure 6B). The heterogeneity predominantly originated from the LI subgroups, C subgroups and C.T subgroups.

##### 3.5.1.2 AST levels

AST levels exhibited a higher trend in the TSG groups in comparison with the control groups. A meticulous subgroup analysis exposed a significant surge in AST levels within the LI [n = 162, 95% *CI* (0.61,1.75), *SMD* = |1.18|, *I* ^*2*^ = 60.9%, *P-value* < 0.0001], C [n = 164, 95% *CI* (0.19,1.28), *SMD* = |0.73|, *I* ^*2*^ = 63.3%, *P-value* = 0.009], and C.T [n = 36, 95% *CI* (0.06,3.82), *SMD* = |1.94|, *I* ^*2*^ = 78.7%, *P-value* = 0.043] subgroups. Conversely, no substantial distinction was unearthed in the remaining groups when the intervention groups were appraised against the control groups [N subgroups: n = 126, 95% *CI* (-0.29,0.41), *SMD* = |0.06|, *I* ^*2*^ = 0.0%, *P-value* = 0.721; T subgroups: n = 88, 95% *CI* (-0.23,0.61), *SMD* = |0.19|, *I* ^*2*^ = 0.0%, *P-value* = 0.382] (Figures 6C, D). The LI, C, and C.T subgroups were identified as the primary sources of increased heterogeneity in the subgroup analysis.

##### 3.5.1.3 TNF-α levels

In contrast to the control groups, TNF-α levels were increased by TSG in intervention groups. This increase was noted across both the LI [n = 152, 95% *CI* (0.28,1.41), *SMD* = |0.84|, *I* ^*2*^ = 61.6%, *P-value* = 0.003] and the N [n = 120, 95% *CI* (0.15,1.26), *SMD* = |0.71|, *I* ^*2*^ = 53.0%, *P-value* = 0.013] subgroups as illustrated in Figure 6D. A detailed examination of the isomer-based subgroups indicated a rise in TNF-α levels across all categories [C subgroups: n = 166, 95% *CI* (0.38,1.48), *SMD* = |0.93|, *I* ^*2*^ = 63.0%, *P-value* = 0.001; T subgroups: n = 70, 95% *CI* (-0.19,1.08), *SMD* = |0.45|, *I* ^*2*^ = 42.1%, *P-value =* 0.170; C.T subgroups: n = 36, 95% *CI* (-0.33,1.95), *SMD* = |0.81|, *I* ^*2*^ = 60.6%, *P-value* = 0.164], while T subgroups and C.T subgroups did not exhibit statistical significance (Figure 6E).

##### 3.5.1.4 IL-6 levels

**FIGURE 7:**
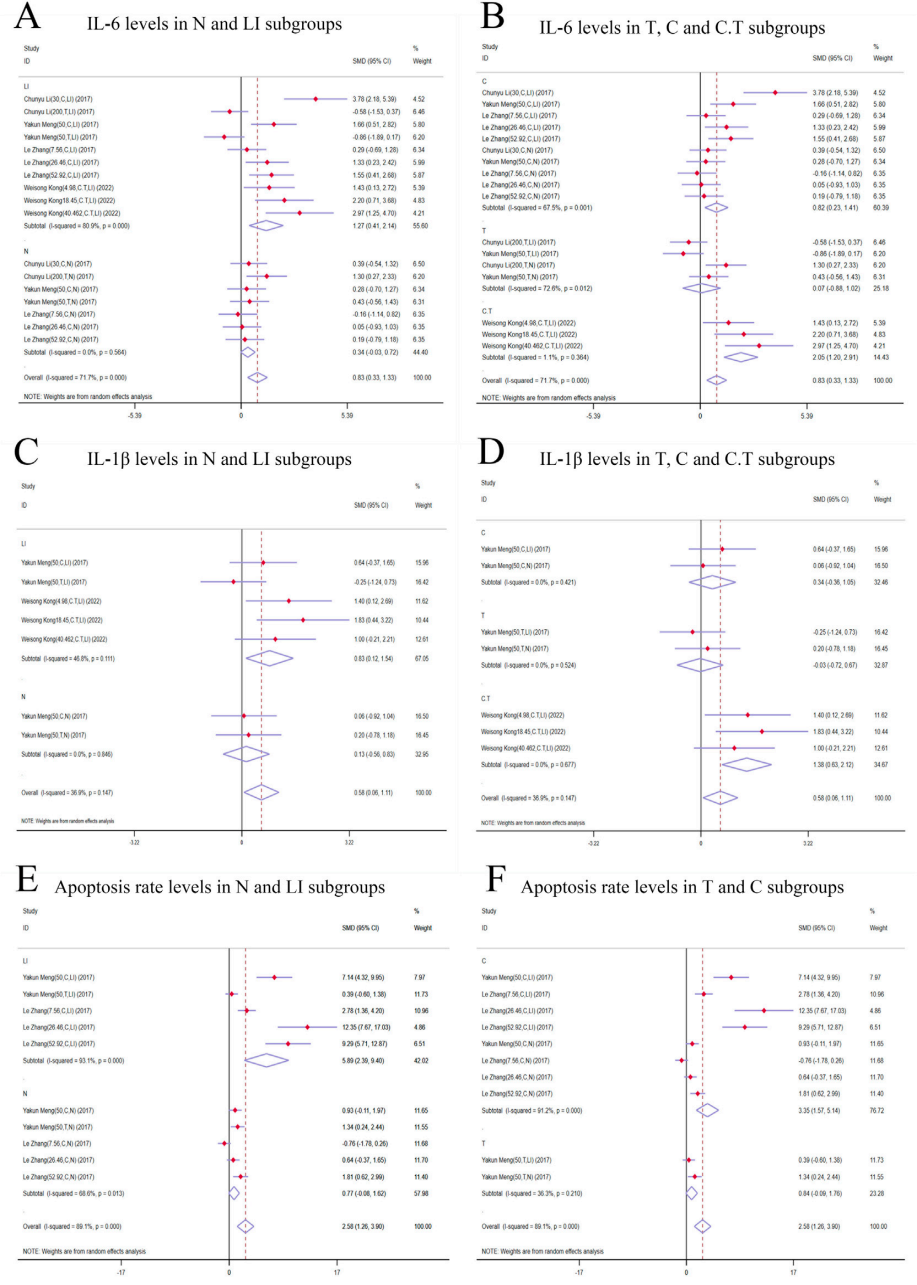
Forest plot (effect size and 95% CI) of cis/trans-SG’s hepatotoxic roles on IL-6, IL-1β and apoptosis rate. **(A)** IL-6 levels in N and LI subgroups; **(B)** IL-6 levels in T, C and C.T subgroups; **(C)** IL-1β levels in N and LI subgroups; **(D)** IL-1β levels in T, C and C.T subgroups; **(E)** Apoptosis rate levels in N and LI subgroups; **(F)** Apoptosis rate levels in T and C subgroups. Abbreviations: 95% CI, 95% confidence interval; IL-6, interleukin 6; IL-1β, interleukin 1β; N, normal; LI, liver injury; T subgroup, trans-SG subgourp; C subgroup, cis-SG subgroup; C.T subgroup, cis-SG and trans-SG subgroup.

Subgroup analysis based on modeling methods and isomers revealed a significant increase in IL-6 levels in LI [n = 152, 95% *CI* (0.41,2.41), *SMD* = |1.27|, *I* ^*2*^ = 80.9%, *P-value* = 0.004], C [n = 164, 95% *CI* (0.23,1.41), *SMD* = |0.82|, *I* ^*2*^ = 67.5%, *P-value* = 0.006], and C.T [n = 36, 95% *CI* (1.20,2.91), *SMD* = |2.05|, *I* ^*2*^ = 1.1%, *P-value* < 0.0001] subgroups. IL-6 levels in N [n = 116, 95% *CI* (-0.03,0.72), *SMD* = |0.34|, *I* ^*2*^ = 0.0%, *P-value* = 0.069] and T [n = 68, 95% *CI* (-0.88,1.02), *SMD* = |0.07|, *I* ^*2*^ = 72.6%, *P-value =* 0.886] subgroups showed no significant difference compared to control groups (Figures 7A, B). Conversely, the N [n = 116, 95% *CI* (-0.03,0.72), *SMD* = |0.34|, *I* ^*2*^ = 0.0%, *P-value* = 0.069] and T [n = 68, 95% *CI* (-0.88,1.02), *SMD* = |0.07|, *I* ^*2*^ = 72.6%, *P-value =* 0.886] subgroups demonstrated no significant deviation in IL-6 levels when compared to their respective control groups (Figures 7A, B).

#### 3.5.2 Secondary indicators of the hepatotoxic effects of cis-SG and trans-SG

##### 3.5.2.1 IL-1β levels

In the subgroup analysis based on modeling methods, IL-1β levels in the LI subgroups were higher than those in the control groups [n = 68, *95% CI* (0.12,1.54), *SMD* = |0.83|, *I* ^*2*^ = 46.8%, *P-value =* 0.022], while no significant difference was found between N subgroups and control groups [n = 32, *95% CI* (-0.56,0.83), *SMD* = |0.13|, *I* ^*2*^ = 0.0%, *P-value =* 0.711] (Figure 7C). Additionally, IL-1β levels exhibited an increasing trend in the C.T subgroups [n = 36, *95% CI* (0.63,2.12), *SMD* = |1.38|, *I* ^*2*^ = 0.0%, *P-value* <0.0001]. The T [n = 32, *95% CI* (-0.72,0.67), *SMD* = |-0.03|, *I* ^*2*^ = 0.0%, *P-value =* 0.942] and C [n = 32, *95% CI* (-0.36,1.05), *SMD* = |0.34|, *I* ^*2*^ = 0.0%, *P-value =* 0.338] subgroups did not show statistical significance (Figure 7D).

##### 3.5.2.2 Apoptosis rate

Apoptosis rate significantly was increased in the LI [n = 80, *95% CI* (2.39,9.40), *SMD* = |5.89|, *I* ^*2*^ = 93.1%, *P-value =* 0.001] and C [n = 128, *95% CI* (1.57,5.14), *SMD* = |3.35|, *I* ^*2*^ = 91.2%, *P-value* <0.0001] subgroups. In contrast, the N [n = 80, *95% CI* (-0.08,1.62), *SMD* = |0.77|, *I* ^*2*^ = 68.6%, *P-value* = 0.078] and T [n = 32, *95% CI* (-0.09,1.76), *SMD* = |0.84|, *I* ^*2*^ = 36.3%, *P-value* = 0.076] subgroups demonstrated no significant changes in apoptosis rates (Figures 7E, F).

### 3.6 Sensitivity analysis and publication bias of outcome indicators

The ability of ALT and AST levels to LI in rodent models was found to be comparably effective. In order to assess potential publication bias, we employed the absolute value of the t-statistic and performed Egger’s test. The absolute t-values for both of these biomarkers did not suggest the presence of publication bias within the included studies (ALT in hepatoprotection, |t|-value = |-3.49|; AST in hepatoprotection, |t|-value = |-3.67|; ALT in hepatotoxicity, |t|-value = |1.07|; AST in hepatotoxicity, |t|-value = |1.2|) (Supplementary Tables 2, 3).

### 3.7 Dose–time–effect/toxicity relationship and machine learning

#### 3.7.1 Effective dose and time length of TSG on ALT and AST levels

**FIGURE 8:**
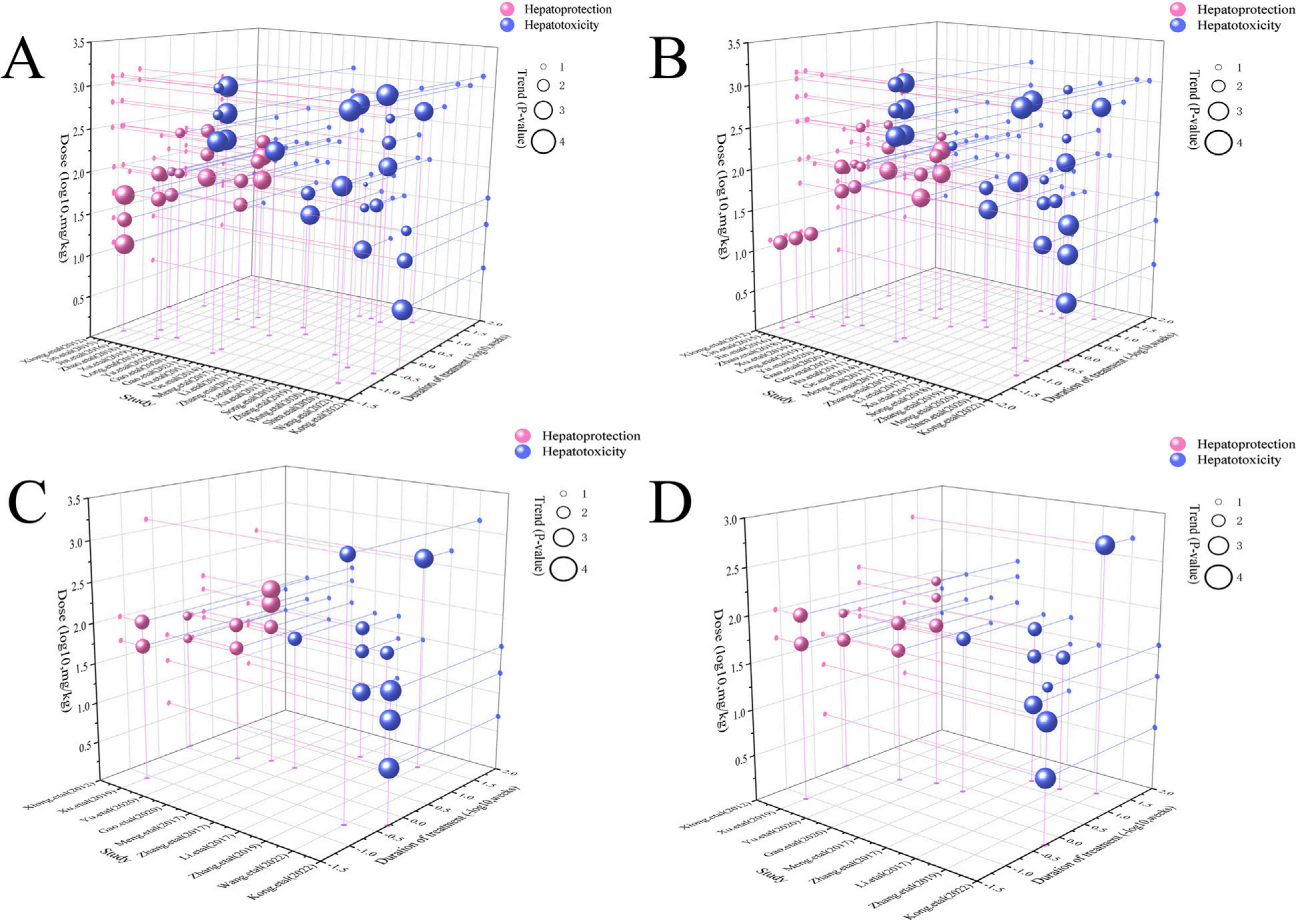
3D maps of dose–time–effect relationship. **(A)** ALT levels; **(B)** AST levels; **(C)** TNF-α levels; **(D)** IL-6 levels.

**FIGURE 9:**
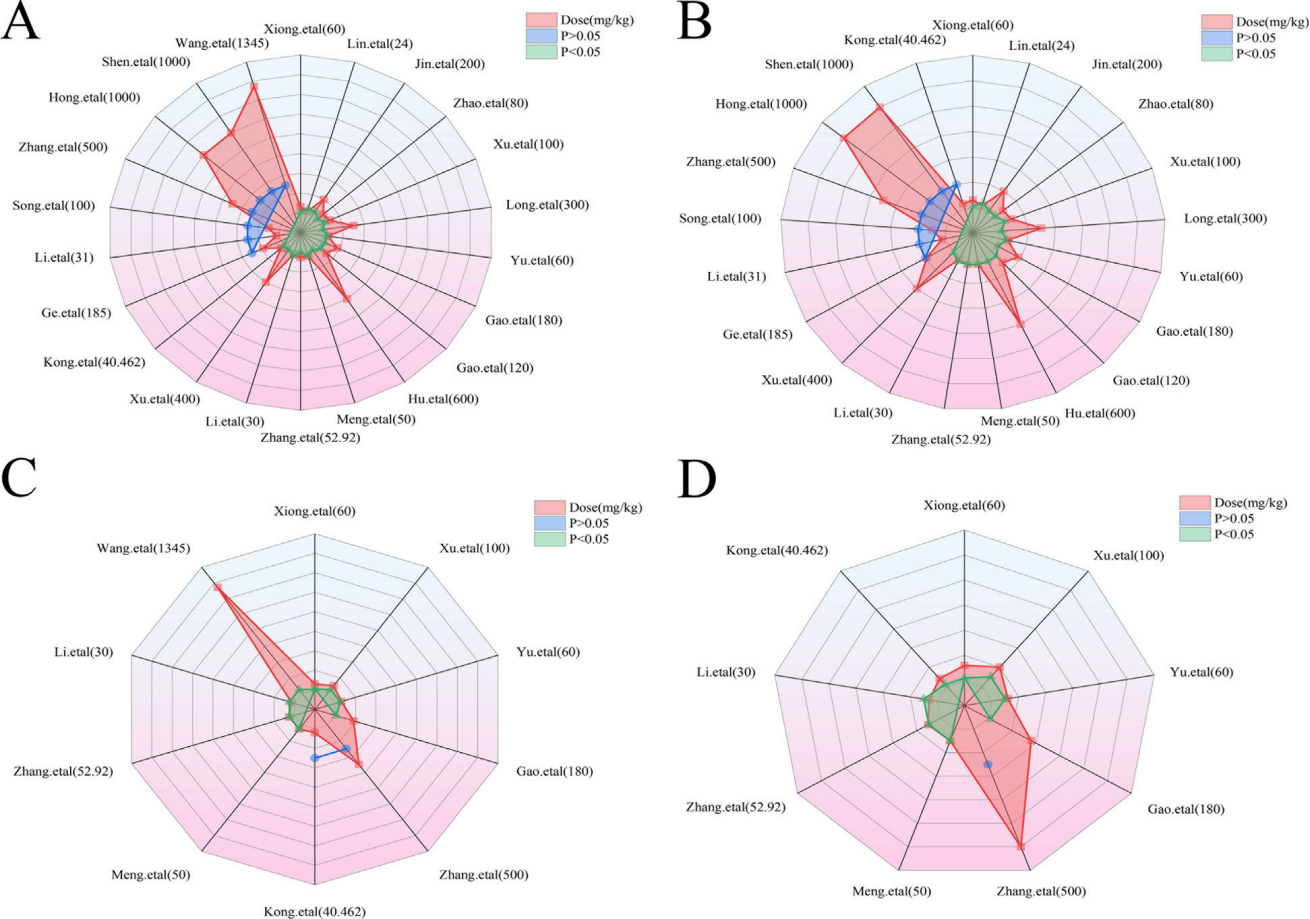
Radar charts of dose–time–effect relationship. **(A)** ALT levels; **(B)** AST levels; **(C)** TNF-α levels; **(D)** IL-6 levels.

**FIGURE 10:**
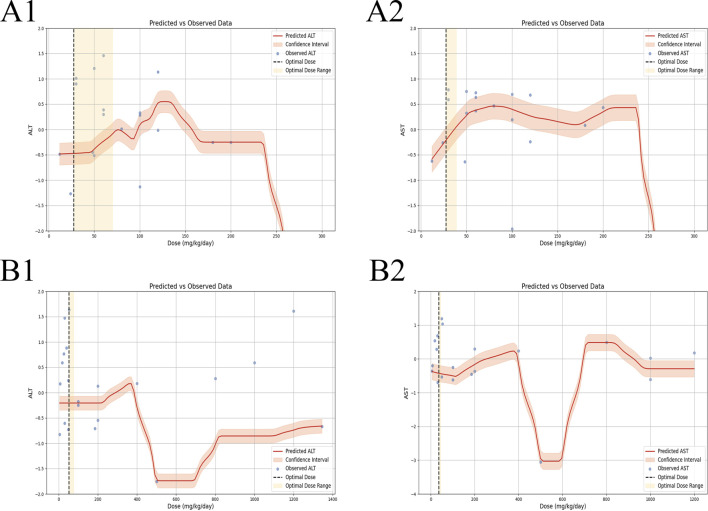
Machine learning of dose–time–effect relationship. **(A1)** ALT levels in hepatoprotection; **(A2)** AST levels in hepatoprotection; **(B1)** ALT levels in hepatotoxicity; **(B2)** AST levels in hepatotoxicity.

In the context of LI models, the therapeutic substance (TSG) has been observed to lower ALT and AST levels when administered at dosages between 30 mg/kg/day and 100 mg/kg/day, as determined by three-dimensional (3D) scatter plot analysis. By employing machine learning techniques, the precise effective dosage was refined to a narrower range of 27.27 mg/kg/day to 38.81 mg/kg/day, with an optimal dosage identified at 27.27 mg/kg/day. It is important to note that these beneficial effects are not present if the dosage falls below the threshold of 27.27 mg/kg/day. In terms of treatment duration, 3D mapping and radar chart analysis suggest that TSG’s efficacy in reducing ALT and AST levels is observed within a window of 0.43 weeks–1 week. Further research is necessary to ascertain the precise dosage and effectiveness of TSG for treatment periods extending beyond 1 week, as depicted in Figures 8–10.

##### 3.7.1.1 Toxic dose and time length of TSG on ALT and AST levels

In the case of toxic effects, TSG has been found to elevate ALT and AST levels in LI models when given at higher dosages, ranging from 50 mg/kg/day to 200 mg/kg/day, as per 3D scatter plot analysis. Machine learning algorithms have pinpointed a more precise toxic dosage range of 51.93 mg/kg/day to 76.07 mg/kg/day, with a maximum toxic effect at 51.93 mg/kg/day. Interestingly, no toxic effects were detected in normal (N) models even at much higher dosages, from 100 mg/kg/day to 1,345 mg/kg/day. In terms of treatment duration, the 3D mapping and radar chart analysis indicate that TSG’s toxicity, as measured by increased ALT and AST levels, is evident within a timeframe of 0.06 weeks–0.43 weeks. The impact of TSG at treatment durations shorter than 0.04 weeks or longer than 12.86 weeks remains unclear and requires further investigation to determine the specific toxic dosage levels of TSG *in vivo*, as illustrated in Figures 8–10.

##### 3.7.1.2 Effective dose and time length of TSG on TNF-α and IL-6 levels

In the context of LI models, the therapeutic substance (TSG) has demonstrated the ability to lower the inflammatory markers TNF-α and IL-6 when administered at daily doses spanning from 30 mg/kg to 60 mg/kg. This finding is contingent upon maintaining all other experimental parameters at their ideal states, with the exception of TSG’s dosage. To pinpoint the precise dosage threshold for TSG’s efficacy, further inquiry is warranted. It’s important to highlight that the reduction in TNF-α and IL-6 levels is not observed at doses below the 30 mg/kg threshold. Regarding the temporal aspect of treatment, three-dimensional graphical representations and radar charts indicate that TSG’s efficacy in modulating TNF-α and IL-6 levels is observed within a period of 0.4–0.86 weeks. Further research is necessary to delineate the optimal dosage of TSG and to assess its impact for treatment durations that surpass 0.86 weeks, as indicated in Figures 8–10.

##### 3.7.1.3 Toxic dose and time length of TSG on TNF-α and IL-6 levels

In the realm of LI models, an increase in TNF-α and IL-6 levels is associated with TSG administration at higher doses, specifically at 50 mg/kg/day, 200 mg/kg/day, 400 mg/kg/day, and 800 mg/kg/day. However, in normal (N) models, no adverse effects of TSG were detected within the dosage range of 26.46 mg/kg/day to 52.92 mg/kg/day. When examining the time frame of treatment, three-dimensional mapping and radar charts reveal that TSG notably elevates TNF-α and IL-6 levels within a span of 0.06–0.43 weeks. Further exploration is essential to establish the exact toxic dosage levels of TSG and to understand its *in vivo* administration effects, as depicted in Figures 8–10.

### 3.8 Network pharmacology of TSG in LI

#### 3.8.1 The common targets and TSG-LI network diagram

**FIGURE 11:**
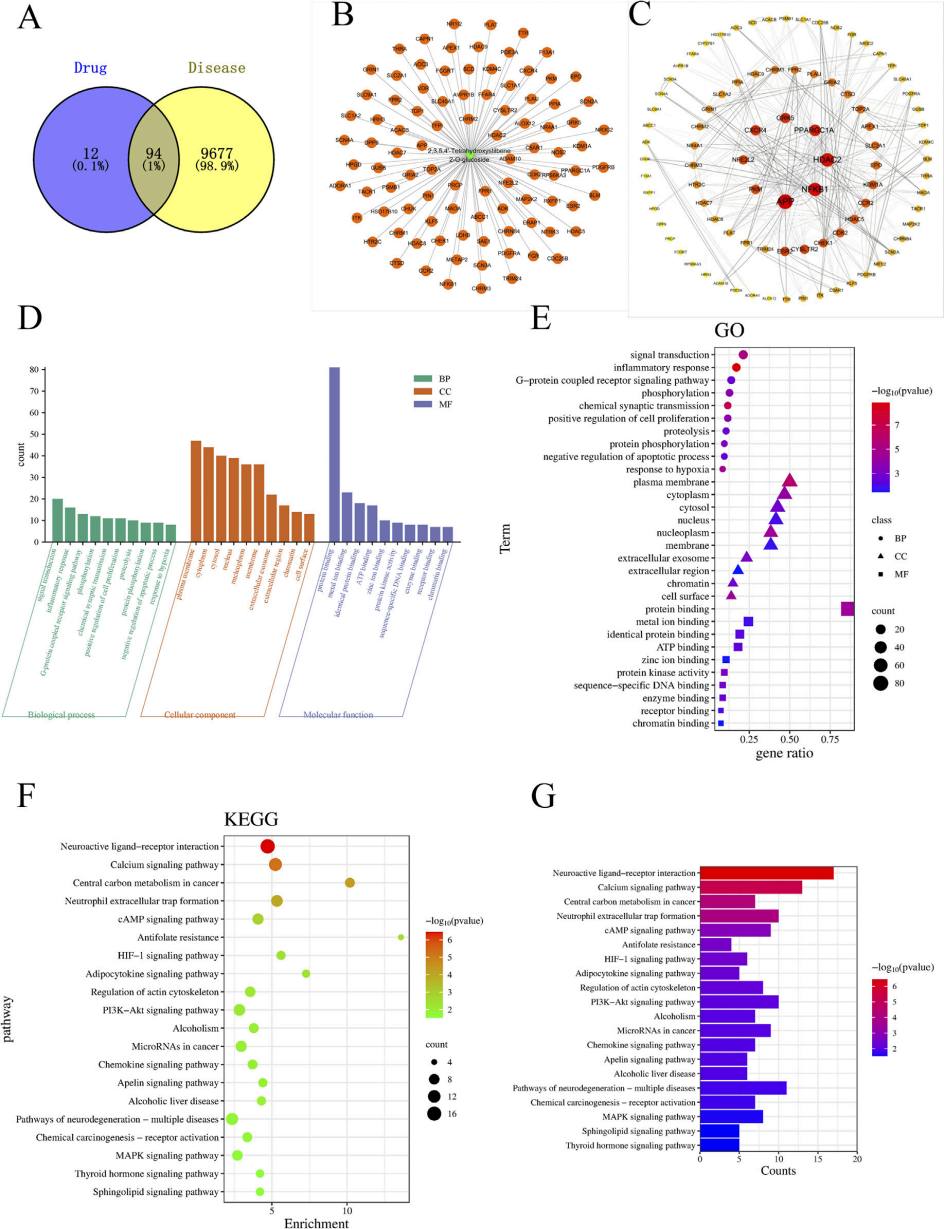
The charts of network pharmacology. **(A)** Venn diagram; **(B)** TSG-LI network diagram; **(C)** PPI network; **(D, E)** Go analysis; **(F, G)** KEGG analysis.

A total of 106 TSG targets were identified after the elimination of duplicates, sourced from the SuperPred and BATMAN databases. Concurrently, 9,700 and 101 LI-associated targets were extracted from the GeneCards and OMIM databases. Uniprot database was used to convert gene names into Symbol IDs. The commonality of active targets between the two conditions was graphically represented in a Venn diagram. The Venn diagram showed that there were 94 common targets between TSG and LI, accounting for 1% (Figure 11A). Following this, the active targets from TSG were incorporated into Cytoscape 3.7.2, resulting in the formation of a drug-ingredient-target network diagram, which consisted of 107 nodes and 106 edges. The CHRM2, HDAC2, ADAM10, NFE2L2, FPR1, PRCP, TOP2A, APP, TFPI and NFE2L2 emerged as central targets within this network (Figure 11B).

#### 3.8.2 The PPI network diagram

After intersecting all TSG-related targets with the target genes of LI, 94 intersection target genes associated with LI and TSG were obtained, representing the interactive target genes between the drug and LI. These 94 intersection target genes were imported into the String (https://string-db.org/) database for protein-protein interaction prediction, with the species set to: *Homo Sapiens* and the confidence level set to: 0.4. The network file was saved in TSV format, and the TSV file was imported into Cytoscape 3.8.2 to draw the protein interaction network, which includes 89 nodes and 460 edges. A topological analysis of the network was conducted, where the degree value was used to indicate the size and color of the targets, as well as the combined score determined the thickness of the edges, thus constructing the protein-protein interaction network as depicted in the illustration. Notably, the nodes with the highest degree of connectivity, ranking in the top 8, included APP, HDAC2, NFKB1, PPARGC1A, CXCR4, GRK5, PKM, and NFE2L2 (Figure 11C).

#### 3.8.3 Go analysis and KEGG pathway enrichment analysis

Employing the DAVID database, we conducted a Gene Ontology (GO) analysis on the intersecting targets, revealing 103 BPs, 44 CCs, and 49 MFs with significant statistical enrichment (*P* < 0.05). The leading five BPs identified were signal transduction, inflammatory response, G-protein coupled receptor signaling pathways, phosphorylation, chemical synaptic transmission, positive regulation of cell proliferation, proteolysis, protein phosphory lation, negative regulation of apoptotic process, and response to hypoxia. In terms of CC, the most prominent were the plasma membrane, cytoplasm, cytosol, nucleus, nucleoplasm, membrane, extracellular exosome, extracellular region, chromatin, and cell surface. For MF, the top categories were protein binding, metal ion binding, identical protein binding, ATP binding, zinc ion binding, protein kinase activity, sequence-specific DNA binding, enzyme binding, receptor binding, and chromatin binding (Figures 11D, E).

For the enrichment of signaling pathways, the DAVID database was again utilized, identifying 40 pathways associated with TSG and LI. With a stringent P-value cutoff of <0.05, 32 pathways were selected as pertinent to the TSG-LI interaction. The top 20 of these pathways were neuroactive ligand-receptor interaction, calcium signaling pathway, central carbon metabolism in cancer, neutrophil extracellular trap formation, cAMP signaling pathway, antifolate resistance, HIF-1 signaling pathway, adipocytokine signaling pathway, regulation of actin cytoskeleton, PI3K-AKT signaling pathway, alcoholism, microRNAs in cancer, chemokine signaling pathway, apelin signaling pathway, alcoholic liver disease, pathways of neurodegeneration-multiple diseases, chemical carcinogenesis-receptor activation, MAPK signaling pathway, thyroid hormone signaling pathway, and sphingolipid signaling pathway (Figures 11F, G).

### 3.9 Potential mechanisms and molecular docking of key targets

The intricate and diverse mechanisms by which TSG influences the progression of LI are not straightforward. Supplementary Tables 8, 9 offers an assessment of the signaling transduction pathways that have been pinpointed, specifically Keap1/Nrf2/HO-1/NQO1, NF-κB, PPAR, as well as TGF-β pathways.

To substantiate the possible mechanisms through which TSG exerts its effects, we employed molecular docking techniques to evaluate the binding affinity of TSG with its principal targets. Our comprehensive molecular docking analysis has unveiled the intimate interactions of TSG with PPARGC1A, NFE2L2, NFKB1, and STAT, complemented by a meticulous examination of the thermodynamic data.

The calculated free energy of −5.1 kcal/mol indicates a strong interaction between TSG and key residues on the PPARGC1A protein, including THR215, THR216, TYR213, LYS212, and GLU209. Similarly, a free energy of −5.9 kcal/mol suggests robust binding of TSG to ARG503, ARG502, ARG499, LYS506, and ASN482 on the NFE2L2 protein. TSG also demonstrates substantial binding with NFKB1, highlighted by a free energy of −6.7 kcal/mol, involving residues such as PHE225, THR122, GLU117, ILE120, TYR163, ARG161, and GLY162. Additionally, TSG is shown to have significant interactions with the STAT protein, with an estimated free energy of −6.7 kcal/mol, engaging residues ASN662, GLU618, ASP627, HIS629, GLN621, and PRO626. These interactions are characterized by hydrogen bonding and hydrophobic contacts.

**FIGURE 12:**
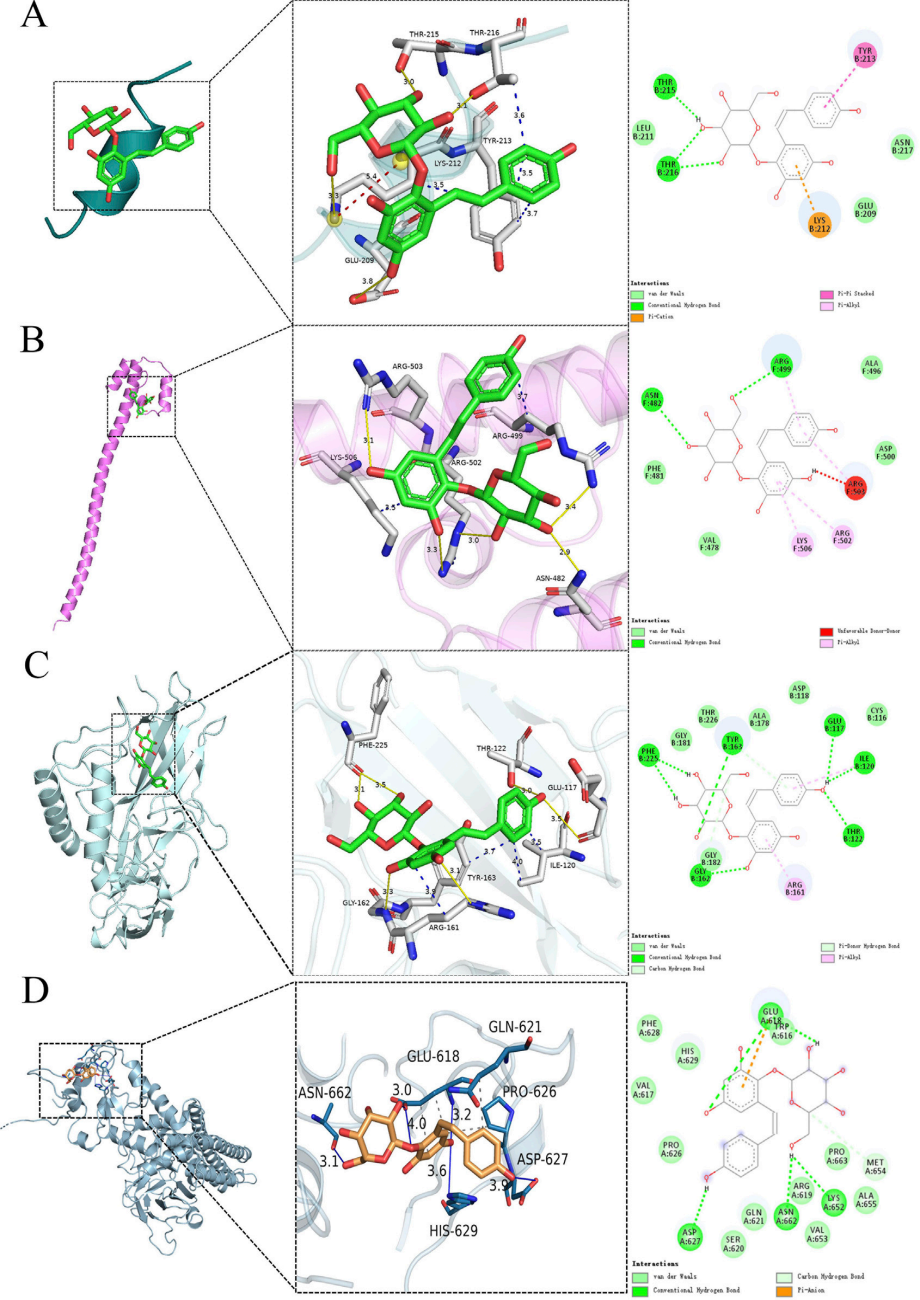
Molecular docking of TSG and key targets. **(A)** TSG binding to PPARGC1A; **(B)** TSG binding to NFE2L2; **(C)** TSG binding to NFKB1; **(D)** TSG binding to STAT.

The visualization of the compound-target interactions was accomplished using PyMoL 2.6 and Discovery Studio 2019 (Figure 12). This study provides a comprehensive view of the molecular interactions that underpin the biological activity of TSG, offering insights into their potential therapeutic applications.

## 4 Discussion

**FIGURE 13:**
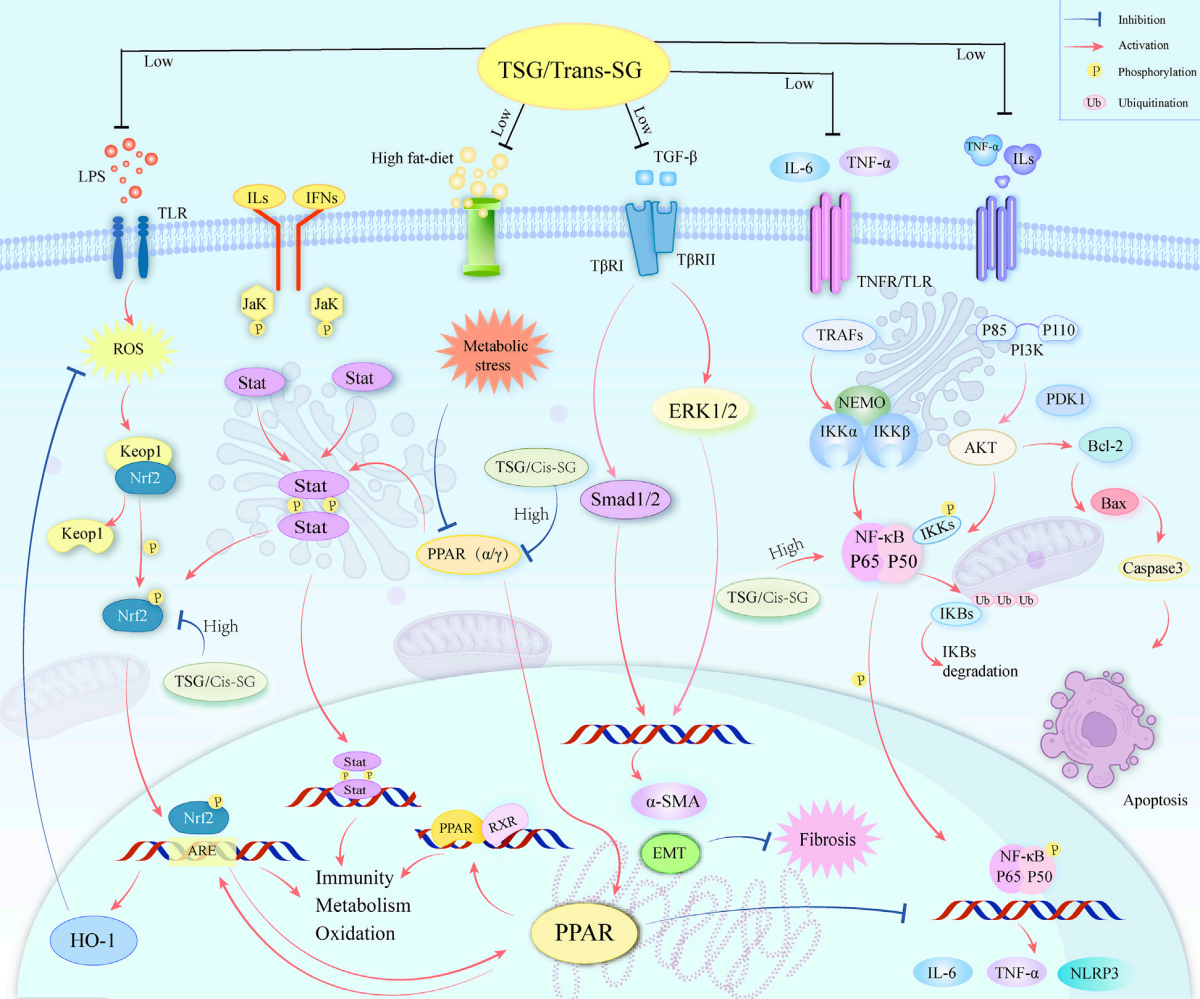
A graphical representation illustrates multiple molecular processes of TSG’s protective and toxic in liver injury by modifying Keap1/Nrf2/HO-1/NQO1, NF-κB, PPAR, PI3K/Akt, TGF-β/Smad, TGF-β/ERK, as well as JAK/STAT signaling pathways.

TSG, a bioactive substance originating from the plant *P. multiflorum* Thunb., has garnered considerable attention for its dual influence on LI. Our study encompassed 24 scholarly articles that featured 564 rodent subjects, highlighting TSG’s role in both liver protection and liver damage. We scrutinized a spectrum of biomarkers, such as ALT, AST, TNF-α, IL-6, serum TG, serum TC, SOD, MDA, IFN-γ, and the apoptosis rate, to assess the therapeutic efficacy and the dosage sensitivity of TSG in addressing both the reparative and harmful aspects of LI. Furthermore, we endeavored to elucidate the underlying mechanisms of TSG’s protective and toxic effects by employing network pharmacology and molecular docking (Figure 13).

### 4.1 Protective mechanisms of TSG on LI

The protective effects of TSG on LI have been a focal point of research, given its multifaceted regulatory roles in various signaling pathways. As elucidated through literature and network pharmacology, TSG is posited to mediate its protective effects by intricately regulating a spectrum of pathways. These include the Keap1/Nrf2/HO-1/NQO1, NF-κB, PPAR, PI3K/Akt, transforming growth factor beta (TGF-β)/small mothers against decapentaplegic (Smad), as well as TGF-β/extracellular signal-regulated kinase (ERK) pathways. The modulation of these pathways is pivotal in mitigating the levels of liver enzymes, such as ALT and AST, which are indicative of LI. TSG’s capacity to diminish these enzyme levels is instrumental in alleviating liver damage.

Furthermore, TSG has been observed to attenuate the levels of key inflammatory markers, including TNF-α and IL-6, which are associated with LI. It also modulates the rate of apoptosis by reducing the expression of pro-apoptotic factors and enhancing the expression of anti-apoptotic factors, thereby diminishing cell death in the liver (Wang et al., 2020a; Chen et al., 2020). This multifaceted action underscores TSG’s potential as a therapeutic agent in the management of liver health.

The PI3K/Akt pathway, a critical signaling mechanism extensively studied for its role in cell survival and liver protection, is activated by TSG (Khezri et al., 2022; Li and Wang, 2014; Wang et al., 2020b). This activation leads to the phosphorylation of Akt, a serine/threonine kinase integral to fostering cell survival and averting apoptosis (Li and Wang, 2014; Lin et al., 2024). In the context of LI, TSG’s activation of the PI3K/Akt pathway has been shown to bolster hepatocyte survival, thus contributing to liver repair and regeneration. Specifically, TSG treatment has been reported to activate the PI3K/Akt pathway, inducing autophagy in the liver, which serves a protective role against prediabetic injury by curbing inflammation and cell death while promoting cell proliferation (Qian et al., 2021; Wang et al., 2020b). In research conducted on human neuroblastoma cell lines (SH-SY5Y), it was discovered that the TSG enhances cell survival and reduces the likelihood of programmed cell death, or apoptosis, by increasing the levels of phosphatidylinositol 3-kinase (PI3K), protein kinase B (Akt), and the survival-promoting protein Bcl-2, while simultaneously decreasing the levels of the pro-apoptotic protein Bax (Kang et al., 2024). This indicates that TSG’s protective effect against cell death may be mediated through the PI3K/Akt signaling pathway. TSG’s influence on the expression levels of caspase 3, Bcl-2-associated X protein (Bax), and B-cell lymphoma-2 (Bcl-2) is significant, as these proteins play a pivotal role in the regulation of apoptosis. Bcl-2 is known for its anti-apoptotic properties, whereas Bax is associated with promoting cell death (Glick et al., 2010; Zhang et al., 2024a). The equilibrium between these proteins is critical in the determination of whether cells live or die, and TSG’s ability to modulate their expression suggests its potential to influence the cellular milieu towards a pro-survival state and against cell death by apoptosis (Atmaca et al., 2024; Wang et al., 2024a).

The PI3K/Akt signaling pathway interacts with the NF-κB signaling pathway, with the two pathways mutually regulating each other and jointly participating in a variety of biological processes, especially in cell survival, inflammatory responses, and immune responses (Aksoy et al., 2005; Li et al., 2024; Shafiek et al., 2024; Yu et al., 2024). The PI3K/Akt signaling pathway can activate the NF-κB signaling pathway by phosphorylating IκB kinase α (IKKα), thereby promoting the release of NF-κB from the IκB complex in the cytoplasm, and subsequently migrating to the nucleus to activate the expression of related genes (Agarwal et al., 2005; Aksoy et al., 2005; Bai et al., 2009; Oeckinghaus et al., 2011). In addition, Akt can activate the NF-κB pathway by phosphorylating IκB kinase (IKK), thereby affecting cell survival, proliferation, invasion, angiogenesis, and chemotherapy resistance (Agarwal et al., 2005; Bai et al., 2009). However, some studies have reported that the NF-κB pathway can be inhibited by upregulating the phosphorylation of PI3K and Akt while reducing the phosphorylation of IκBα and NF-κB, thereby inhibiting inflammatory responses (Agarwal et al., 2005; Misra et al., 2006). TSG may also have a similar pathway, inhibiting inflammatory responses by suppressing the PI3K/Akt/NF-κB pathway, which requires experimental validation.

Inflammation and immune response are critical components of LI (Jia et al., 2024; Wang et al., 2024b), and TSG modulates these through the inhibition of NFKB1. NF-κB is a vital transcription factor involved in immune responses, inflammation, cell growth, and stress responses (Hayden and Ghosh, 2011; Hoesel and Schmid, 2013; Lawrence, 2009; Yu H. et al., 2020). It is typically inactive in the cytoplasm due to interaction with IκB proteins. Upon receipt of pro-inflammatory signals, a signaling cascade is initiated that activates the IκB kinase (IKK) complex, leading to the degradation of IκB and the release of NF-κB into the nucleus to regulate gene expression (Lim et al., 2019). In LI, therapeutic substances like TSG may protect the liver by reducing the production of pro-inflammatory cytokines such as TNF-α and IL-6 and modulating NF-κB signaling (Lin et al., 2015b). They could potentially diminish NF-κB’s nuclear translocation by inhibiting IκB phosphorylation and degradation, reducing inflammatory factor expression (Ma et al., 2016; Xiong et al., 2012). TSG might also regulate antioxidant stress response proteins to prevent NF-κB activation caused by oxidative stress. Understanding NF-κB’s relationship with oxidative stress is crucial for developing treatments for diseases where oxidative stress is a factor (Deng et al., 2024; Ma et al., 2024; Yu H. et al., 2020).

Oxidative stress, a state caused by an imbalance between oxidation and antioxidation within the body, leads to the production of a plethora of oxidative intermediates that can damage cellular structures and affect their physiological functions (Filomeni et al., 2015; Sies, 2015; Thomas et al., 2024). The NF-κB pathway has both antioxidant and pro-oxidant roles in the context of oxidative stress. Reactive oxygen species (ROS) can activate or inhibit NF-κB signaling in a context-dependent manner (Morgan and Liu, 2011). There is a crosstalk between NF-κB and the Nrf2 signaling pathway, which is involved in the response to antioxidative stress (Oeckinghaus et al., 2011). During the oxidative stress response, electrophilic metabolites inhibit the activity of the BCR (Keap1) complex, promoting the formation of heterodimers between Nrf2 and small Maf proteins, which then accumulate in the nucleus (Bellezza et al., 2018). The activation of Nrf2 can regulate a series of genes involved in antioxidation and metabolic detoxification, and the activity of Nrf2 is also regulated by NF-κB (Oeckinghaus et al., 2011).

TSG exerts a multifaceted influence on LI, primarily through the modulation of oxidative stress and the antioxidant response. It triggers the activation of Nrf2, a key controller of cellular antioxidant processes, leading to an upregulation of genes responsible for detoxification and antioxidant production, such as those for heme oxygenase (HO-1) and quinone oxidoreductase-1 (NQO1) (Gao et al., 2020; Li et al., 2020; Loboda et al., 2016; Yu W. et al., 2020). This enhancement of the liver’s ability to counteract reactive oxygen species (ROS) and preserve cellular redox equilibrium is crucial in reducing a primary cause of LI. Under normal physiological conditions, the activity of Nrf2 is controlled by Keap1, a protein rich in cysteine residues that marks Nrf2 for proteasomal degradation via the Cul3-dependent E3 ubiquitin ligase pathway (Baird and Yamamoto, 2020; Bellezza et al., 2018). However, under stress, Nrf2 is phosphorylated, enabling its release from Keap1, nuclear translocation, and subsequent binding to Maf proteins (Bellezza et al., 2018). This binding event initiates the activation of the antioxidant response element (ARE), which drives the transcription of genes that Nrf2 regulates, playing a central role in the cellular response to oxidative stress, including anti-inflammatory, antioxidant, and apoptotic activities (Jia et al., 2024; Katsuoka et al., 2005; Ulasov et al., 2022). TSG’s involvement in the Nrf2/HO-1 signaling axis is particularly noteworthy in lessening the impact of acetaminophen (APAP)-induced LI (Gao et al., 2020), where it helps to alleviate lipid peroxidation and metabolic disturbances, underscoring its potential as a therapeutic agent for liver health.

TSG also plays a significant role in LI protection by modulating the TGF-β signaling pathway, central to the development of hepatic fibrosis (Long et al., 2019). By inhibiting the phosphorylation of key pathway components like ERK1/2 and Smad1/2 (Peng et al., 2022), TSG can attenuate the fibrotic response characterized by excessive extracellular matrix deposition and tissue scarring. This modulation is further supported by TSG’s ability to suppress inflammation, promote liver regeneration, and reduce the activation of hepatic stellate cells, pivotal in fibrosis. Additionally, TSG’s influence on immune responses could indirectly affect the TGF-β pathway, potentially protecting hepatocytes by curbing inflammation and oxidative stress. This may interfere with the TGF-β activation and its downstream signaling, including the Smad-dependent and Smad-independent pathways, which involve the activation of ERK and its role in cell survival and epithelial–mesenchymal transition (EMT) (Hata and Chen, 2016; Peng et al., 2022). Overall, TSG’s intervention in the TGF-β/Smad and TGF-β/ERK pathways presents a promising therapeutic strategy for LI management (Long et al., 2019), aiming to regulate gene expression involved in cell proliferation, differentiation, and matrix production (Peng et al., 2022; Zhang et al., 2017).

The liver, as the metabolic command center for lipids, dutifully orchestrates the synthesis, secretion, and clearance of cholesterol and lipoproteins, which are the circulatory workhorses for lipids (Nguyen et al., 2008). TSG has been recognized for its ability to ignite the peroxisome proliferator-activated receptor alpha (PPARα) signaling pathway (Xu et al., 2019), a conductor of lipid metabolism and guardian of cellular homeostasis (Bougarne et al., 2018; Wang et al., 2020). Within the context of a meticulous study probing the effects of TSG on rats befallen by fatty liver disease, courtesy of a high-fat diet, the application of TSG was lauded for its capacity to significantly curtail the levels of total cholesterol, triglycerides, and free fatty acids in both serum and liver tissue (Xi, 2018; Xu et al., 2019). This salutary effect was found to be in tandem with an upregulation of PPARα and the autophagy-associated proteins LC3II and Beclin 1, while simultaneously orchestrating a retreat for p62 (Xi, 2018). The TSG-induced activation of autophagy is theorized to embolden the disintegration of lipid droplets, thereby refining the liver’s lipid metabolic prowess and elevating its overall metabolic acumen.

In summary, TSG’s intricate action on molecular targets results in a multifaceted response to LI, providing a comprehensive protective mechanism against liver damage. The precise mechanisms are still under investigation, but it is believed that TSG modulates various pathways, from the cell membrane to the nucleus, influencing the transcription of genes related to inflammation, fibrosis, and liver regeneration. This integrated approach, which enhances antioxidant defenses, regulates metabolism, curbs inflammation, and affects signal transduction, underscores TSG’s potential as a therapeutic agent in liver disease management.

### 4.2 Hepatotoxic mechanisms of TSG on LI

TSG is a natural compound found in the dried root of *P. multiflorum* Thunb., and exhibits both hepatoprotection and hepatotoxicity. The molecular signaling pathways involved in TSG-induced hepatotoxicity are complex and multifaceted including proliferator-activated receptor (PPAR), JAK (Janus Kinase)/STAT (Signal Transducers and Activators of Transcription), Keap1/Nrf2/HO-1, and NF-κB signaling pathways.

The hepatotoxicity of TSG is deeply interwoven with its impact on energy metabolism and mitochondrial function, essential for liver health maintenance. TSG targets the PPARGC1A gene, which encodes the transcriptional coactivator PGC-1α, pivotal in regulating genes involved in energy metabolism (Liang and Ward, 2006; Tiraby and Langin, 2005). In synergy with PPARγ, this coactivator enhances mitochondrial gene expression, promoting energy production through fatty acid oxidation and oxidative phosphorylation, a critical mechanism for alleviating metabolic stress on the liver, especially during injury (Christofides et al., 2021; Wang et al., 2020). This process is also considered a key regulator of gluconeogenesis and adaptive thermogenesis (Hosseini et al., 2024; Tiraby and Langin, 2005).

However, TSG’s hepatotoxic effects are manifested through the inhibition of PPARGC1A and PPARγ expression, thereby activating the NF-κB and STAT signaling pathways, commonly dysregulated in liver diseases and potentially leading to LI (Kong et al., 2022; Meng et al., 2017; Zhang, 2017). PPARs, a group of nuclear receptors, significantly regulate cellular processes such as differentiation, development, metabolism, and inflammatory responses (Berthier et al., 2021). Notably, PPAR-γ activation has demonstrated a protective role against LI, potentially through the inhibition of the NF-κB pathway (Shishodia et al., 2007; Zhang, 2017). In LI cases induced by Polygonum multiflorum Thunb., PPAR-γ expression levels have been negatively correlated with the extent of LI, suggesting that PPAR-γ agonists could counteract the LI caused by this traditional medicine (Meng et al., 2017).

The PPAR pathway is intricately involved with multiple pathways, and studies have observed that PPARγ activation can synergize with the Nrf2 pathway, promoting the expression of related genes and inhibiting ferroptosis (Reuter et al., 2010). The activation of the PPAR signaling pathway can also foster the anti-inflammatory differentiation of macrophages in a JAK2/STAT6 dependent manner, simultaneously activating both PPARγ and Nrf2 signals (Tu et al., 2023). This suggests a potential interaction between PPAR and Nrf2, highlighting their joint role in regulating inflammatory and antioxidant responses (Ghanim and Qinna, 2022; Tu et al., 2023). Furthermore, the PPAR pathway intersects with the NF-κB pathway, playing a significant role in modulating inflammatory responses and cellular metabolism (de Souza Basso et al., 2021; Reuter et al., 2010). These complex interactive networks may contribute to the hepatotoxicity induced by TSG.

The JAK/STAT pathway is a pivotal mechanism for intracellular communication, playing a role in numerous biological functions such as cellular proliferation, maturation, and immune system reactions (Hu et al., 2021; Xin et al., 2020). Dysfunctional activation of this pathway has been linked to a range of illnesses, including those affecting the liver (Owen et al., 2019; Xin et al., 2020). Research has shown that disruptions in the JAK/STAT signaling are prevalent in liver conditions associated with Hepatitis B Virus (HBV), influencing both the onset and progression of these diseases (Tang et al., 2023; Xu et al., 2022; Zhou et al., 2021). Moreover, an overactive JAK/STAT signaling pathway is a significant factor in the development and worsening of hepatocellular carcinoma, potentially serving as a key biomarker for assessing the severity and predicting the outcome of this type of liver cancer (Lokau et al., 2019; Zhao et al., 2021). Although these studies do not directly point out the mechanism by which TSG produces liver toxicity through the JAK/STAT pathway, they provide a connection between the JAK/STAT pathway and liver diseases. Additionally, a study based on an *in vitro* hepatotoxicity assessment system using liver organoids and high-content imaging technology has differentiated the hepatotoxic potential of TSG and its cis-isomer (cis-SG) in Polygonum multiflorum (Liu et al., 2022). It was found that the hepatotoxicity of cis-SG is related to mitochondrial damage, and this hepatotoxicity can be inhibited by mitochondrial protective agents. This suggests that some isomers of TSG may affect mitochondrial function, thereby affecting the JAK/STAT signaling pathway, leading to liver cell damage. Although there is currently no direct evidence to show the detailed mechanism by which TSG produces liver toxicity through the JAK/STAT pathway, we can speculate that TSG may affect the JAK/STAT pathway based on existing research and molecular docking results, thereby affecting the function and survival of liver cells, ultimately leading to liver toxicity. Future research needs to further explore the specific mechanism of TSG’s impact on the JAK/STAT pathway and how to mitigate or prevent LI caused by TSG by regulating this pathway.

TSG has garnered attention for its possible role in intensifying LI via the Keap1/Nrf2/HO-1 axis. This axis is a significant protective system against oxidative stress and plays a crucial role in both the prevention and mitigation of LI (Liu et al., 2022). Typically, Nrf2 is marked for degradation by the Keap1-CUL3 complex through ubiquitination, but when stress is present, Nrf2 detaches from Keap1, accumulates in the cytoplasm, and then moves to the nucleus to bind with specific genes, thereby triggering the transcription of genes that encode for antioxidant and detoxification enzymes (Ghanim and Qinna, 2022; Liu et al., 2022). In scenarios of TSG-induced hepatotoxicity, it has been noted that TSG can boost the expression and activity of CYP450 enzymes, which are key in the metabolism of drugs into potentially harmful reactive metabolites that can trigger LI (Manikandan and Nagini, 2018). Notably, TSG has been linked to the upregulation of CYP2E1, CYP3A4, and CYP1A2, which could lead to an increased metabolic conversion of hepatotoxic substances and a worsening of LI (Xu et al., 2017). Additionally, TSG has been observed to trigger the nuclear translocation of the aryl hydrocarbon receptor (AHR) and the pregnane X receptor (PXR), both of which are involved in the regulation of CYP1A2 and CYP3A4 expression (Meng, 2021; Xu et al., 2017). The suppression of AHR or PXR by specific inhibitors has been shown to lessen the exacerbating effect of TSG on acetaminophen-induced hepatotoxicity, suggesting that these transcription factors play a part in TSG’s influence on LI (Meng, 2021). All in all, while TSG possesses various beneficial pharmacological properties, it also has the potential to induce hepatotoxicity by modulating the Keap1/Nrf2/HO-1 pathway and increasing the expression of CYP450 enzymes, which could enhance the metabolic activation of hepatotoxic compounds. The precise mechanisms of TSG’s impact on the Keap1/Nrf2/HO-1 pathway and its role in LI require further investigation to fully understand its hepatotoxic potential and to develop strategies for the safe use of TSG-containing herbal remedies.

The NF-κB pathway is often activated in liver diseases and can contribute to LI when persistently activated (Liu et al., 2017). TSG has been reported to trigger the proliferation of CD4^+^ T and CD8^+^ T cells and the secretion of cytokines *in vivo*, suggesting its potential to initiate an immune response that may contribute to LI (Liu et al., 2024). The activation of T cells and the secretion of inflammatory cytokines such as TNF-α and IFN-γ can lead to the activation of the NF-κB pathway. Once activated, NF-κB can translocate to the nucleus and promote the transcription of genes involved in inflammation and cell survival (Lawrence, 2009). The exact mechanisms of TSG-induced hepatotoxicity through the NF-κB pathway are not fully understood. TSG may contribute to hepatotoxicity potentially through the NF-κB pathway by modulating the immune response and potentially interacting with other hepatotoxic compounds.

The PPAR, Nrf2, JAK/STAT, and NF-κB pathways are intricately linked and may all be implicated in LI induced by TSG. These pathways form an interactive network that is centered around the PPAR pathway. The PPAR/JAK/STAT/Nrf2 axis stands out as a crucial component of this network. Upon activation by their respective ligands, PPARs can modulate the expression of target genes, including those involved in the JAK/STAT pathway (Das et al., 2024). Once activated, JAKs phosphorylate STAT proteins, enabling them to dimerize and translocate to the nucleus, where they act as transcription factors regulating gene expression (Morris et al., 2018). Activated STAT proteins can then interact with Nrf2, which, when stabilized and activated, translocates to the nucleus and binds to antioxidant response elements (AREs), inducing the expression of detoxifying and antioxidant enzymes (Wang and He, 2022).

Furthermore, PPARs can interact with Nrf2 to produce synergistic effects, such as antioxidant actions (Reuter et al., 2010). The activation of PPARγ enhances the expression and activity of Nrf2, which in turn further stimulates the transcription of antioxidant and detoxifying enzymes (Abdelhamid et al., 2020; Zhang et al., 2018). This mutual promotion between PPAR and Nrf2 strengthens the cell’s defense mechanisms against oxidative stress and other forms of cellular injury, highlighting their integral role in maintaining liver health and their potential as therapeutic targets for liver diseases (Zhang et al., 2018). TSG may inhibit Nrf2 activity by suppressing the PPAR/JAK/STAT/Nrf2 axis, while simultaneously activating NF-κB, contributing to LI. The crosstalk between these pathways and their combined impact on LI induced by TSG underscores the complexity of the hepatic response to this compound and suggests that interventions targeting this network could be beneficial in ameliorating liver damage.

In summary, the PPAR/JAK/STAT/Nrf2 axis, along with the NF-κB pathway, forms a complex regulatory network that plays a significant role in TSG-induced LI. TSG may cause LI through various mechanisms, including negative impacts on energy metabolism and mitochondrial function, activation of pathways related to inflammation and immune responses, and enhancement of oxidative stress. These findings emphasize the need for further research into the hepatotoxic mechanisms of TSG and the development of strategies to mitigate or prevent LI caused by TSG.

### 4.3 Hepatotoxic mechanisms of cis-SG/trans-SG on LI

Cis-SG and trans-SG are two isomers found in the dried root of *P. multiflorum* Thunb., commonly known as *Heshouwu*. They exhibit different mechanisms of hepatotoxicity. Cis-SG has demonstrated a stronger hepatotoxicity compared to trans-SG *in vivo* experiments. Studies indicate that cis-SG may cause liver damage by affecting multiple molecular signaling pathways. For instance, cis-SG may affect the function of mitochondria, leading to cellular energy metabolism disorders. Specifically, cis-SG may cause an increase in mitochondrial membrane permeability, leading to a decrease in mitochondrial membrane potential (MMP), thereby triggering mitochondrial dysfunction (Liu et al., 2022). Additionally, cis-SG can downregulate the expression of PPAR-γ, activate the NF-κB signaling pathway, and induce monocytes/macrophages to secrete pro-inflammatory cytokines such as TNF-α and IL-6, leading to liver damage (Zhang, 2017). In contrast, trans-SG has not been observed to have significant hepatotoxic effects under normal administration conditions. However, if phase II metabolism is inhibited during the metabolic process, the risk of liver damage from trans-SG may increase. *In vitro* experiments have shown that trans-SG mainly undergoes phase II metabolism through UGT enzymes, and its metabolites are glucuronic acid conjugates (Li N. et al., 2017). When phase II metabolic enzymes are inhibited using ketoconazole, the degree of LI caused by trans-SG in LPS-sensitized rat models significantly increases, indicating that the metabolic state of trans-SG may significantly impact its risk of liver damage (Li N. et al., 2017).

It is worth noting that the hepatotoxicity of trans-SG and cis-SG in *Heshouwu* may have synergistic effects with other components, and LI caused by *Heshouwu* may involve various mechanisms, including immune stress, oxidative stress, and endoplasmic reticulum stress (Liang et al., 2024). Therefore, although cis-SG plays a major role in LI caused by *Heshouwu*, the metabolism and interactions of trans-SG and other components may also adversely affect the liver under certain conditions. To elaborate further, the hepatotoxicity mechanisms of trans-SG and cis-SG involve intricate cellular processes. Cis-SG, being more hepatotoxic, can disrupt cellular homeostasis by interacting with specific receptors and triggering a cascade of responses that lead to inflammation and cell death. On the other hand, trans-SG’s impact is less pronounced unless metabolic pathways are compromised, leading to the accumulation of potentially toxic metabolites.

The dose-time-toxicity relationship is crucial in understanding the hepatotoxic potential of these compounds. The severity of LI is not only dependent on the concentration of these isomers but also on the duration of exposure. Continuous or high-dose exposure to cis-SG can lead to more significant liver damage, whereas trans-SG may only pose a risk under conditions that inhibit its metabolism.

The hepatotoxicity of trans-SG and cis-SG is a multifactorial process involving complex molecular signaling pathways and is influenced by dosage and exposure time. Further research is necessary to fully elucidate the mechanisms and identify potential therapeutic strategies to mitigate the hepatotoxic effects of these compounds in *Heshouwu*.

### 4.4 The dual effects of TSG depend on dosage and subgroups analysis

This study included 564 animals for meta-analysis, confirming the hepatotoxicity and hepatoprotective effects of TSG. In terms of hepatoprotective effects, TSG significantly reduced the levels of ALT, AST, TNF-α, IL-6, MDA, Serum TG, and Serum TC, while increasing the levels of SOD and GSH. The therapeutic effect of TSG on LI showed no significant differences across BI, NBI, Rats, and Mice subgroups, all significantly reducing the levels of main indicators. However, in terms of hepatotoxicity, TSG significantly increased the levels of ALT, AST, TNF-α, IL-6, IFN-γ, and Apoptosis rate. Due to the large differences in the levels of hepatotoxicity indicators across groups, we conducted further subgroup analyses. The results confirmed that TSG has obvious hepatotoxicity in the LI model subgroup and rat subgroup, while no obvious hepatotoxicity was found in the N model subgroup and mice. TSG also has two isomers (cis-SG and trans-SG), therefore we conducted subgroup analyses of the hepatotoxicity of the two isomers separately. The results showed that cis-SG could significantly increase the indicators and has obvious hepatotoxicity, while trans-SG showed no significant toxicity. Although trans-SG has not been found to exhibit significant hepatotoxicity, the levels of LI indicators are further increased when cis-SG is used in combination with trans-SG.

In order to develop and apply the drug, it is essential to reduce the toxic effects while ensuring the efficacy of the drug. We used machine learning, 3D scatter plots, and radar charts to divide the dose range of TSG that causes hepatotoxicity and hepatoprotection. The results show that the optimal dose range for TSG to treat LI is from 27.27 mg/kg/d to 38.81 mg/kg/d, with the best dose being 27.27 mg/kg/d. The optimal dose range for TSG to cause LI is from 51.93 mg/kg/d to 76.07 mg/kg/d, with the best dose being 51.93 mg/kg/d. Trans-SG, due to its therapeutic effect on LI and relatively low toxic side effects, may be the direction for drug development.

### 4.5 Limitations

The present article strictly followed the PRISMA guidelines, albeit with certain inherent limitations. Here are the refined points: 1. The study’s scope was confined to a selection of four English and four Chinese databases, which inevitably introduced a degree of selectivity bias. Furthermore, it was not feasible to encompass the entire body of pertinent literature. 2. The diversity across the studies was challenging to fully reconcile, due to factors such as discrepancies in measurement tools, unit variances, and experimental design differences. 3. The study’s corpus was limited to peer-reviewed articles, excluding reviews, correspondence, conference papers, and theses. 4. While articles with quality scores below the threshold of 5 were systematically excluded, the potential for result heterogeneity persists due to the variable quality of the included studies. 5. The lack of standardization in animal intervention protocols, dosages, treatment schedules, and model species across studies significantly contributed to the observed heterogeneity. 6. The research validated the potency and dependability of TSG in addressing liver impairment or hepatotoxic conditions by conducting a sensitivity analysis, applying Egger’s test, and performing subgroup analyses, thereby bolstering the trustworthiness of the outcomes. 7. Although the study encapsulated the principal therapeutic mechanisms of TSG in safeguarding the liver and inducing hepatotoxic effects, a complete overview of every mechanism was not feasible due to the complexity inherent in the pathophysiological processes involved. 8. Ethical considerations have restricted the availability of literature on TSG’s toxicological effects in humans, leading to an exclusive focus on animal model studies. The necessity for clinical trials to validate TSG’s clinical utility in hepatoprotection and hepatotoxicity management is underscored. 9. Although molecular docking provided initial validation of TSG’s interaction with key proteins, further experimental validation is essential for definitive conclusions. 10. TSG can cause various organ injuries, such as liver injury and kidney injury, but several articles reporting the toxicity of TSG mainly focus on its hepatotoxicity, with only a small number of studies reporting its nephrotoxicity. Therefore, this article only focuses on the hepatotoxicity of TSG.

Despite these constraints, the study’s findings have the potential to inform novel clinical strategies and contribute to the advancement of pharmaceutical development.

## 5 Conclusion

TSG’s protective role against LI is attributed to its ability to decrease ALT and AST levels through multiple pathways, including Keap1/Nrf2/HO-1/NQO1, NF-κB, PPARα, PI3K/Akt, and TGF-β/Smad, as well as TGF-β/ERK pathways. These effects are observed at dosages ranging from 27.27 mg/kg/d to 38.81 mg/kg/d and over a period of 0.43 weeks–1 week. Conversely, at higher dosages between 51.93 mg/kg/d and 76.07 mg/kg/d and within the time of 0.06 weeks–0.43 weeks, TSG can increase ALT and AST levels through pathways associated with PPAR, JAK/STAT, Keap1/Nrf2/HO-1, and NF-κB, potentially leading to LI. It is important to note that hepatotoxicity induced by TSG is only evident in LI models and not observed in N models. In comparative *in vivo* studies, cis-SG has exhibited a more pronounced hepatotoxic effect compared to its isomer, trans-SG. Interestingly, trans-SG has shown negligible hepatotoxicity, indicating a significant difference in the biological activity of these isomers.

## Data Availability

The original contributions presented in the study are included in the article/Supplementary Material, further inquiries can be directed to the corresponding authors.

## Glossary

ALT: Alanine aminotransferase
AST: Aspartate aminotransferase
ALP: Alkaline phosphatase
ALB: Albumin
Akt: Protein kinase B
ARE: Antioxidant-responsive elements
APAP: Acetaminophen
BI: Biomacromolecule induced
Bax: Bcl-2-associated X protein
Bcl-2: B-cell lymphoma-2
C subgroup: Cis-SG subgroup
CC: Cellular component
Cul3: Cullin3
CYP450: Cytochrome p450
C.T subgroup: Cis-SG and trans-SG subgroup
DILI: Drug-induced liver injury
ERK: Extracellular signal-regulated kinase
EASL: European Association for the Study of the Liver
GSH: Glutathione
HFD: High-fat die
HO-1: Heme oxygenase-1
I: Intervention
**I** ^**2**^: I-squared
IFN-γ: Interferon gamma
IL-6: Interleukin 6
IL-1β: Interleukin 1β
IKKα: IκB kinase α
ICR mice: Institute of Cancer Research mice
JAK: Janus kinase
Keap1: kelch-like ECH-associated protein 1
LI: Liver injury
MF: Molecular function
MSE: Mean squared error
MDA: Malondialdehyde
MMP: Mitochondrial membrane potential
N: Normal
NBI: Non-biomacromolecule induced
Nrf2: Nuclear factor erythroid 2-related factor 2
NF-κB: Nuclear factor kappa-B
NQO-1: Quinone oxidoreductase-1
PPI: Protein-protein interaction
PI3K: Phosphatidylinositol 3-kinase
PPAR: Peroxisome proliferator-activated receptor
BP: Biological process
ROS: Reactive oxygen species
RBF: Radial Basis Function
SD: Standard deviation
SOD: Superoxide dismutase
SMD: Standardised mean difference
Smad: Small mothers against decapentaplegic
STAT: Signal transducer and activator of transcription
T subgroup: Trans-SG subgroup
TP: Total protein
TC: Total cholesterol
TCM: Traditional Chinese medicine
TG: Triglyceride
TSG: 2,3,5,4′-tetrahydroxystilbene-2-O-β-D-glucopyranoside
TGF-β: Transforming growth factor beta
TNF-α: Tumor necrosis factor alpha
W: Week(s)
95%CI: 95% confidence interval
