# Supplementary material for: Angel or devil: the dual roles of 2,3,5,4′-tetrahydroxystilbene-2-O-β-D-glucopyranoside in the development of liver injury based on integrating pharmacological techniques: a systematic review
Source: Front Pharmacol. 2025 Feb 3;16:1523713. doi: 10.3389/fphar.2025.1523713 (PMC11830817; doi:10.3389/fphar.2025.1523713)
Supplement: Supplementary file 1 [file DataSheet1.pdf]

Supplementary table 1: Retrieval strategy

Number	Search terms
#1	2,3,5,4'-tetrahydroxystilbene 2-O-glucopyranoside [Supplementary Concept]
#2	(2,3,5,4'-tetrahydroxystilbene-2-O-beta-D-glucoside [Title/Abstract]) OR (2,3,5,4'-Tetrahydroxystilbene 2-O-glucoside [Title/Abstract]) OR (2,3,5,4-tetrahydroxyl diphenylethylene-2-o-glucoside [Title/Abstract]) OR (TSG [Title/Abstract]) OR (THSG [Title/Abstract]) OR (Stilbene glucosides [Title/Abstract]) OR (C20H22O9 [Title/Abstract])
#3	(2,3,5,4'-tetrahydroxy-trans-stilbene-2-O-beta-glucoside [Title/Abstract]) OR (trans-SG [Title/Abstract]) OR (trans-stilbene glycoside [Title/Abstract]) OR (trans-2,3,5,4'-tetrahydroxy-stilbene-2-O-β-D-glucoside [Title/Abstract]) OR (trans-2,3,5,4'-tetrahydroxystilbene-2-O-β-D-glucoside [Title/Abstract])
#4	(cis-stilbene glucoside[Title/Abstract]) OR (cis-SG[Title/Abstract]) OR (2,3,5,4'-tetrahydroxy-cis-stilbene-2-O-β-glucoside[Title/Abstract]) OR (cis-2,3,5,4'-tetrahydroxy-stilbene-2-O-β-D-glucoside[Title/Abstract]) OR (cis-2,3,5,4'-tetrahydroxystilbene-2-O-β-D-glucoside[Title/Abstract])
#5	#1 OR #2 OR #3 OR #4
#6	"Liver"[Mesh]
#7	(hepatic injury[Title/Abstract]) OR (liver injury[Title/Abstract]) OR (hepatoprotection[Title/Abstract]) OR (liver fibrosis[Title/Abstract]) OR (hepatic fibrosis[Title/Abstract])
#8	(hepatic injury[Title/Abstract]) OR (liver injury[Title/Abstract]) OR (hepatotoxicity[Title/Abstract]) OR (hepatic toxicity[Title/Abstract]) OR (liver toxicity[Title/Abstract]) OR (liver fibrosis[Title/Abstract]) OR (hepatic fibrosis[Title/Abstract])
#9	#6 OR #7 OR #8
#10	#5 AND #9

Supplementary Table 2: Stratified analysis of main outcome indicators from studies of hepatoprotection of TSG

Indicators	Experiments(n)	Individuals(n)	SMD	95%CI	P-value	Heterogeneity
ALT	18	162	-3.30	(-3.81,-2.80)	P<0.0001	33.5%
AST	18	162	-3.80	(-5.07,-2.53)	P<0.0001	80.6%
TNF-α	8	68	-2.63	(-3.64,-1.62)	P<0.0001	52.3%
IL-6	8	68	-3.44	(-5.23,-1.65)	P<0.0001	80.7%
GSH	10	75	3.61	(1.97,5.26)	P<0.0001	75.1%
MDA	10	82	-2.66	(-3.89,-1.44)	P<0.0001	66.7%
SOD	12	112	2.87	(1.65,4.09)	P<0.0001	74.6%
Serum TG	8	54	-4.14	(-7.15,-1.13)	0.007	86.6%
Serum TC	8	54	-5.40	(-9.19,-1.61)	0.005	87.0%

Supplementary Table 3: Stratified analysis of subgroups of hepatoprotection of TSG

Indicators	Subgroups	Individuals(n)	SMD	95%CI	P-value	Heterogeneity
ALT	BI	86	-3.46	(-4.18,-2.75)	P<0.0001	46.9%
	NBI	76	-3.14	(-3.85,-2.44)	P<0.0001	27.0%
	Rats	34	-4.09	(-5.42,-2.76)	P<0.0001	56.8%
	Mice	128	-3.17	(-3.71,-2.63)	P<0.0001	14.1%
AST	BI	86	-3.25	(-4.25,-2.22)	P<0.0001	52.2%
	NBI	76	-4.88	(-8.27,-1.49)	0.005	90.9%
	Rats	34	-5.97	(-11.05,-0.89)	0.021	84.0%
	Mice	128	-3.55	(-4.93,-2.18)	P<0.0001	82.3%

Supplementary Table 4: Stratified analysis of main outcome indicators from studies of hepatotoxicity of TSG

Indicators	Experiments(n)	Individuals(n)	SMD	95%CI	P-value	Heterogeneity
ALT	26	222	0.88	(0.06,1.69)	0.034	85.3%
AST	24	210	1.03	(0.23,1.82)	0.011	83.5%
TNF-α	12	90	1.24	(0.36,2.11)	0.006	70.0%
IL-6	10	78	1.79	(0.31,3.27)	0.018	85.1%
IFN-γ	6	50	1.41	(0.77,2.05)	P<0.0001	48.7%
Apoptosis rate	6	48	5.64	(0.14,11.14)	0.044	93.6%

Supplementary Table 5: Stratified analysis of subgroups of hepatotoxicity of TSG

Indicators	Subgroups	Individuals(n)	SMD	95%CI	P-value	Heterogeneity
ALT	N	120	0.19	(-1.00,1.38)	0.755	87.7%
	LI	102	1.66	(0.67,2.65)	0.001	76.8%
	Rats	138	1.01	(-0.34,2.36)	0.144	90.1%
	Mice	84	0.63	(-0.08,1.34)	0.083	57.8%
AST	N	108	0.08	(-0.89,1.05)	0.867	81.3%
	LI	102	2.09	(0.92,3.26)	P<0.0001	80.8%
	Rats	138	0.87	(-0.27,2.00)	0.135	87.2%
	Mice	72	1.25	(0.16,2.34)	0.025	74.6%
TNF-α	N	28	0.55	(-1.20,2.29)	0.541	78.1%
	LI	62	1.59	(0.67,2.50)	0.001	57.2%
	Rats	66	1.10	(-0.14,2.34)	0.082	80.2%
	Mice	24	1.58	(0.65,2.52)	0.001	0.0%

Supplementary Table 6: Stratified analysis of main outcome indicators from studies of hepatotoxicity of cis/trans-SG

Indicators	Experiments(n)	Individuals(n)	SMD	95%CI	P-value	Heterogeneity
ALT	38	288	0.59	(0.18,1.00)	0.005	62.6%
AST	38	288	0.65	(0.27,1.04)	0.001	57.5%
TNF-α	34	272	0.78	(0.39,1.17)	P<0.0001	56.0%
IL-6	34	268	0.83	(0.33,1.33)	0.001	71.7%
IL-1β	14	100	0.58	(0.06,1.11)	0.029	36.9%
Apoptosis rate	20	160	2.58	(1.26,3.90)	P<0.0001	89.1%

Supplementary Table 7: Stratified analysis of subgroups of hepatotoxicity of Cis/Trans-SG

Indicators	Subgroups	Individuals(n)	SMD	95%CI	P-value	Heterogeneity
ALT	N	126	0.03	(-0.32,0.38)	0.875	0.0%
	LI	162	1.08	(0.44,1.72)	0.001	69.6%
	T	88	0.09	(-0.33,0.50)	0.689	0.0%
	C	164	0.81	(0.16,1.46)	0.015	72.9%
	C.T	36	1.11	(-0.44,2.67)	0.161	76.2%
AST	N	126	0.06	(-0.29,0.41)	0.721	0.0%
	LI	162	1.18	(0.61,1.75)	P<0.0001	60.9%
	T	88	0.19	(-0.23,0.61)	0.382	0.0%
	C	164	0.73	(0.19,1.28)	0.009	63.3%
	C.T	36	1.94	(0.06,3.82)	0.043	78.7%
TNF-α	N	120	0.71	(0.15,1.26)	0.013	53.0%
	LI	152	0.84	(0.28,1.41)	0.003	61.6%
	T	70	0.45	(-0.19,1.08)	0.170	42.1%
	C	166	0.93	(0.38,1.48)	0.001	63.0%
	C.T	36	0.81	(-0.33,1.95）	0.164	60.6%
IL-6	N	116	0.34	(-0.03,0.72)	0.069	0.0%
	LI	152	1.27	(0.41,2.41)	0.004	80.9%
	T	68	0.07	(-0.88,1.02)	0.886	72.6%
	C	164	0.82	(0.23,1.41)	0.006	67.5%
	C.T	36	2.05	(1.20,2.91）	P<0.0001	1.1%
IL-1β	N	32	0.13	(-0.56,0.83)	0.711	0.0%
	LI	68	0.83	(0.12,1.54)	0.022	46.8%
	T	32	-0.03	(-0.72,0.67)	0.942	0.0%
	C	32	0.34	(-0.36,1.05)	0.338	0.0%
	C.T	36	1.38	(0.63,2.12)	P<0.0001	0.0%
Apoptosis rate	N	80	0.77	(-0.08,1.62)	0.078	68.6%
	LI	80	5.89	(2.39,9.40)	0.001	93.1%
	T	32	0.84	（-0.09,1.76）	0.076	36.3%

C	128	3.35	(1.57,5.14)	P<0.0001	91.2%

Supplementary Table 8: Mechanisms of included studies of TSG in the hepatotoxicity

Category	*Studies*	*Year*	*Proposed mechanisms*
Toxicity	***Zhang.etal***	***2017***	Cis-SG may synergize with LPS by downregulating PPAR - γ expression and activating NF - κB signaling pathway, releasing inflammatory cytokines such as TNF - α and IL-6 to cause liver injury.
Toxicity	***Meng.etal***	***2017***	Cis-SG induces immunological idiosyncratic hepatotoxicity through suppressing PPAR-γ in a rat model of liver injury.
Toxicity	***Xu.etal***	***2017***	TSG exacerbates acetaminophen-induced hepatotoxicity by inducing hepatic expression of CYP2E1, CYP3A4 and CYP1A2.
Toxicity	***Li.etal***	***2017***	Trans-SG causes livery injury through UGT mediated phase II metabolism.
Toxicity	***Song.etal***	***2018***	TSG can disrupt bile acid metabolism and cause liver toxicity by inhibiting MRP2 and inducing BSEP.
Toxicity	***Sun.etal***	***2021***	TSG can disrupt bile acid homeostasis and inhibit phospholipid secretion, causing liver damage. Its mechanism may be related to TSG's inhibition of FXR and downstream targets BSEP and MDR2 expression, as well as disruption of MDR2 distribution in the bile duct membrane.
Toxicity	***Kong.etal***	***2022***	Cis-SG and trans-SG synergistically activate NF-κB signaling in the idiosyncratic induced liver injury.

Supplementary Table 9: Mechanisms of included studies of TSG in the hepatoprotection （

Category	*Studies*	*Year*	*Proposed mechanisms*
Protection	***Xiong.etal***	**2012**	TSG has a protective effect on acute alcoholic liver injury in mice possibly by regulating decreasing endotoxin level, the expression of NF-κB, and IκB-α phosphorylation
Protection	***Lin.etal***	**2015**	The release of downstream inflammatory cytokines in the LPS/TLR4/NF-κB signaling pathway was mitigated by TSG.
Protection	***Jin.etal***	***2016***	TSG can effectively prevent acute alcoholic liver injury as its antioxidant effect and reducing the levels of ALT and AST.
Protection	***Zhao.etal***	***2017***	TSG has a significant therapeutic effect on fatty liver, and its mechanism may be related to its antioxidant effect.
Protection	***Zhao.etal***	***2018***	TSG was effective in treating hyperlipidemic fatty liver in rats. The mechanism may be related to the upregulation of LC3II, Beclin 1, and downregulation of p62 protein expression by activating PPAR α to activate cellular autophagy.
Protection	***Xu.etal***	***2019***	TSG can against non-alcoholic steatohepatitis by regulating key regulators of lipide metabolism (SREBP1c, PPARα and their target genes, ABCG5 and CYP7A1), inflammation (CD68, TNF-α, IL-6 and ICAM), fibrosis (α-SMA and TNF-β) and oxidative stress (NADPH-oxidase 2/4, CYP2E1 and antioxidant enzymes).
Protection	***Long.etal***	***2019***	The beneficial effects of trans-SG are potentially attributed to the inhibition of ERK1/2 and Smad 1/2 phosphorylation in the TGF-β signaling pathway and the blockage of ECM degradation by reducing the expression of MMPs.
Protection	***Yu.etal***	***2020***	TSG can alleviate diethylnitrosamine-induced acute hepatotoxicity by modulating the Nrf2-related antioxidant system and metabolic activation of diethylnitrosamine.
Protection	***Gao.etal***	***2021***	TSG can against liver injury through stimulating Keap1/Nrf2/HO-1/NQO1 signaling pathway.

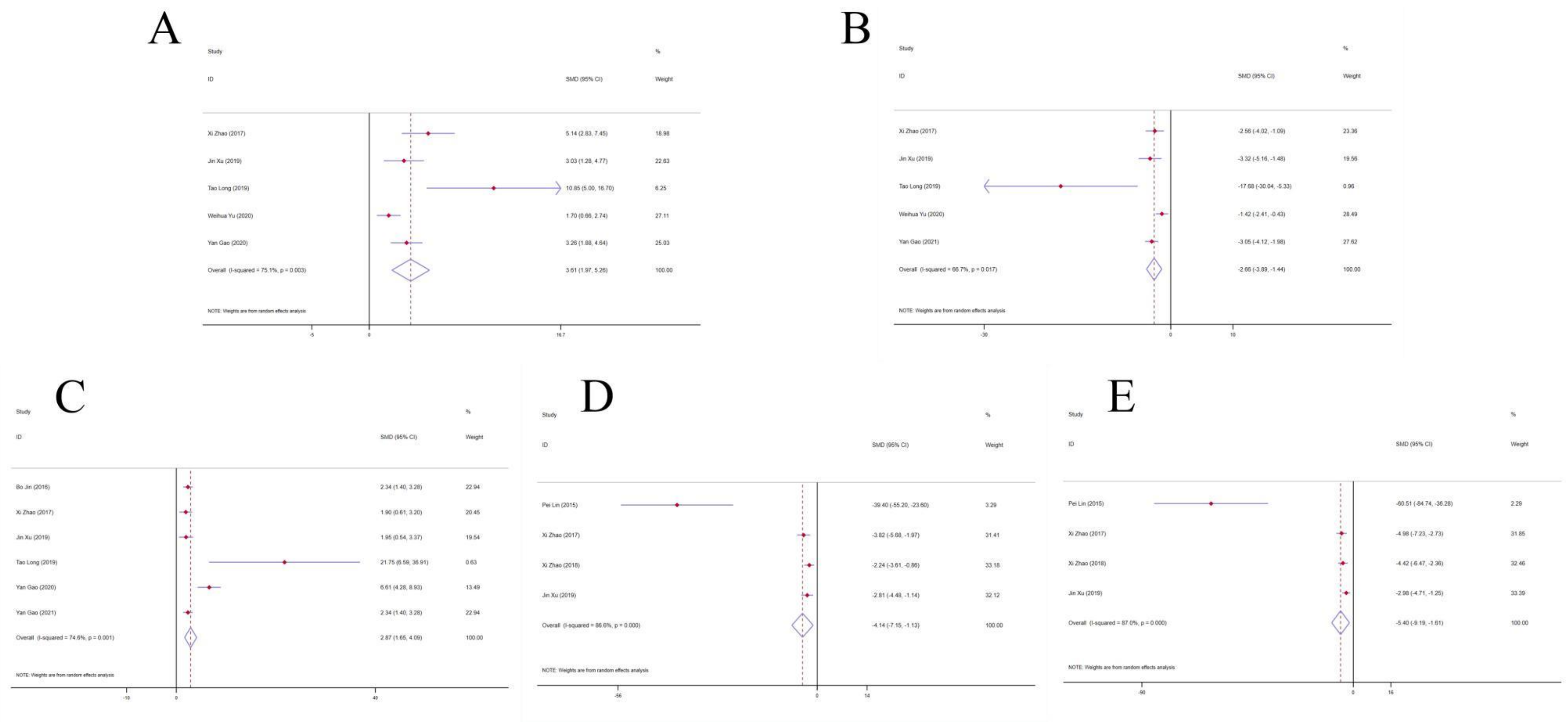

Supplementary Figure 1 Forest plot (effect size and 95% CI) of TSG's hepatoprotective roles on GSH, MDA, SOD, serum TG and serum TC. (A) GSH levels; (B) MDA levels; (C) SOD levels; (D) Serum TG levels; (E) Serum TC levels.

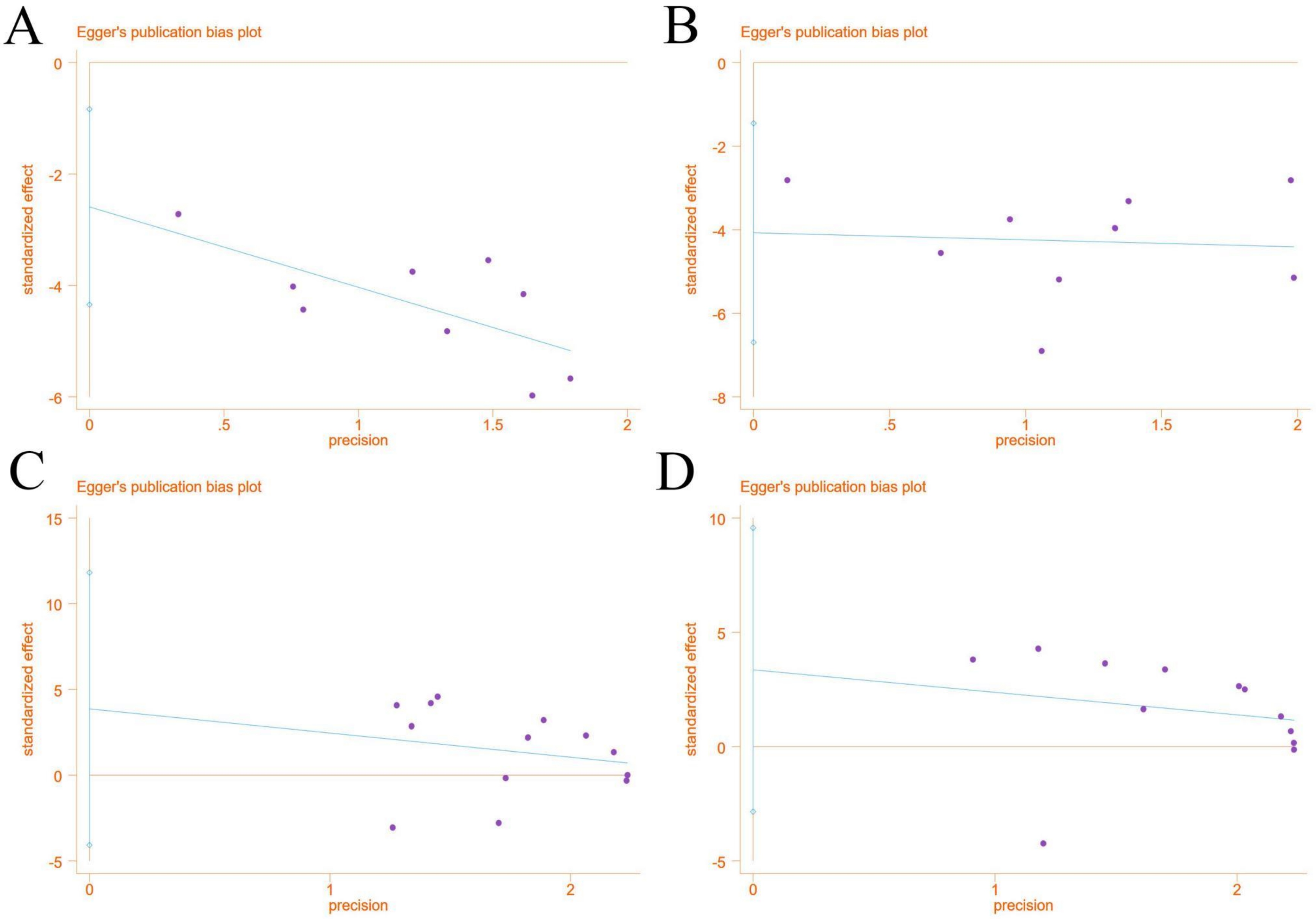

Supplementary figure 2 Egger's publication bias plot. (A)ALT in hepatoprotection, |t| = |-3.49|; (B)AST in hepatoprotection, |t| = |-3.67|; (C)ALT in hepatotoxicity, |t| = |1.07|; (D)AST in hepatotoxicity, |t| = |1.2|.

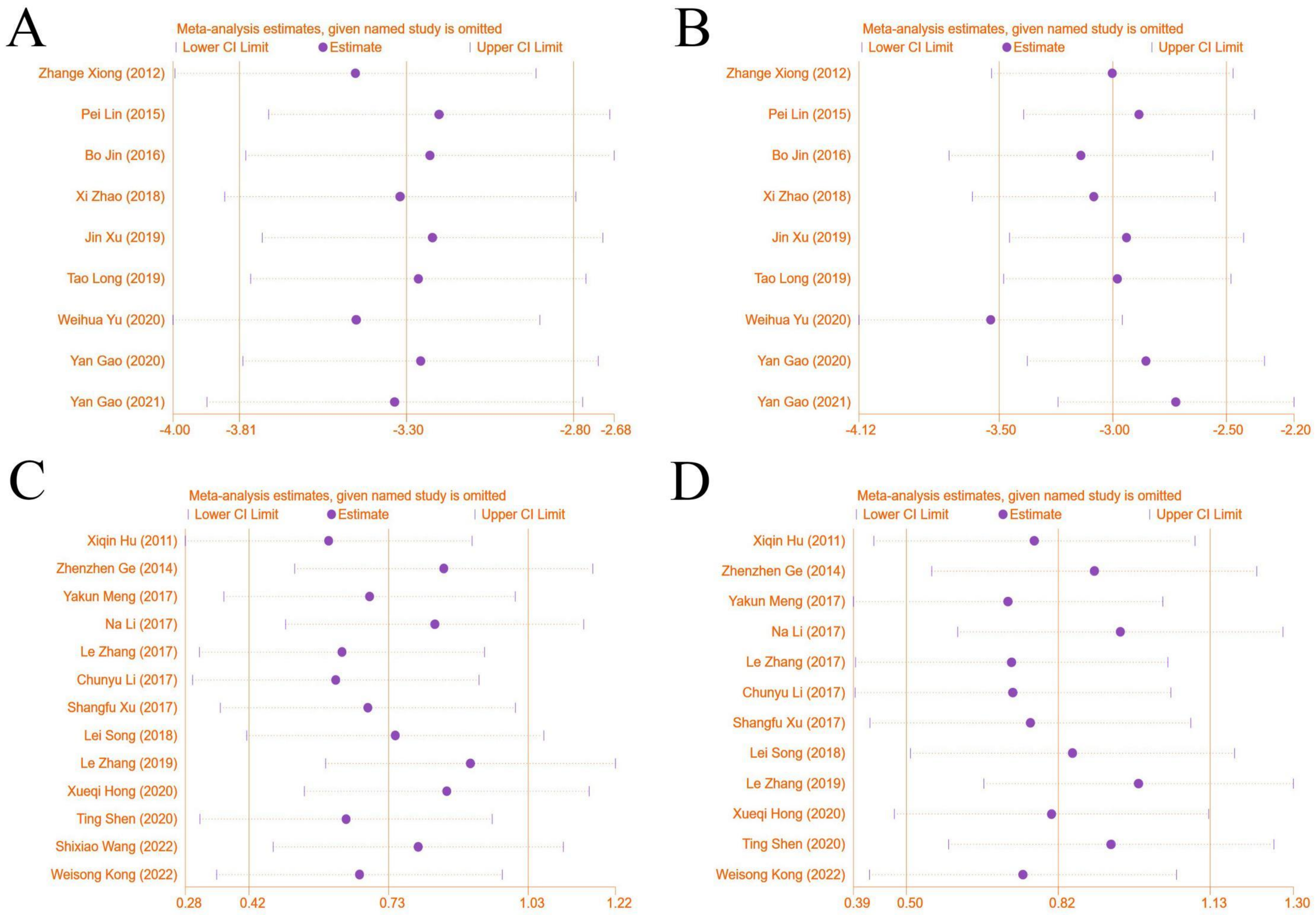

Supplementary figure 3 The plot of Sensitivity test. (A)ALT in hepatoprotection; (B)AST in hepatoprotection; (C)ALT in hepatotoxicity; (D)AST in hepatotoxicity.

PRISMA 2020 Checklist

Section and Topic	Item #	Checklist item	Location where item is reported
**TITLE**			
Title	1	Identify the report as a systematic review.	√
**ABSTRACT**			
Abstract	2	See the PRISMA 2020 for Abstracts checklist.	√
**INTRODUCTION**			
Rationale	3	Describe the rationale for the review in the context of existing knowledge.	√
Objectives	4	Provide an explicit statement of the objective(s) or question(s) the review addresses.	√
**METHODS**			
Eligibility criteria	5	Specify the inclusion and exclusion criteria for the review and how studies were grouped for the syntheses.	√
Information sources	6	Specify all databases, registers, websites, organisations, reference lists and other sources searched or consulted to identify studies. Specify the date when each source was last searched or consulted.	√
Search strategy	7	Present the full search strategies for all databases, registers and websites, including any filters and limits used.	√
Selection process	8	Specify the methods used to decide whether a study met the inclusion criteria of the review, including how many reviewers screened each record and each report retrieved, whether they worked independently, and if applicable, details of automation tools used in the process.	√
Data collection process	9	Specify the methods used to collect data from reports, including how many reviewers collected data from each report, whether they worked independently, any processes for obtaining or confirming data from study investigators, and if applicable, details of automation tools used in the process.	√
Data items	10a	List and define all outcomes for which data were sought. Specify whether all results that were compatible with each outcome domain in each study were sought (e.g. for all measures, time points, analyses), and if not, the methods used to decide which results to collect.	√
	10b	List and define all other variables for which data were sought (e.g. participant and intervention characteristics, funding sources). Describe any assumptions made about any missing or unclear information.	√
Study risk of bias assessment	11	Specify the methods used to assess risk of bias in the included studies, including details of the tool(s) used, how many reviewers assessed each study and whether they worked independently, and if applicable, details of automation tools used in the process.	√
Effect measures	12	Specify for each outcome the effect measure(s) (e.g. risk ratio, mean difference) used in the synthesis or presentation of results.	√
Synthesis methods	13a	Describe the processes used to decide which studies were eligible for each synthesis (e.g. tabulating the study intervention characteristics and comparing against the planned groups for each synthesis (item #5)).	√
	13b	Describe any methods required to prepare the data for presentation or synthesis, such as handling of missing summary statistics, or data conversions.	√

PRISMA 2020 Checklist

Section and Topic	Item #	Checklist item	Location where item is reported
	13c	Describe any methods used to tabulate or visually display results of individual studies and syntheses.	√
	13d	Describe any methods used to synthesize results and provide a rationale for the choice(s). If meta-analysis was performed, describe the model(s), method(s) to identify the presence and extent of statistical heterogeneity, and software package(s) used.	√
	13e	Describe any methods used to explore possible causes of heterogeneity among study results (e.g. subgroup analysis, meta-regression).	√
	13f	Describe any sensitivity analyses conducted to assess robustness of the synthesized results.	√
Reporting bias assessment	14	Describe any methods used to assess risk of bias due to missing results in a synthesis (arising from reporting biases).	√
Certainty assessment	15	Describe any methods used to assess certainty (or confidence) in the body of evidence for an outcome.	√
**RESULTS**			
Study selection	16a	Describe the results of the search and selection process, from the number of records identified in the search to the number of studies included in the review, ideally using a flow diagram.	√
	16b	Cite studies that might appear to meet the inclusion criteria, but which were excluded, and explain why they were excluded.	√
Study characteristics	17	Cite each included study and present its characteristics.	√
Risk of bias in studies	18	Present assessments of risk of bias for each included study.	√
Results of individual studies	19	For all outcomes, present, for each study: (a) summary statistics for each group (where appropriate) and (b) an effect estimate and its precision (e.g. confidence/credible interval), ideally using structured tables or plots.	√
Results of syntheses	20a	For each synthesis, briefly summarise the characteristics and risk of bias among contributing studies.	√
	20b	Present results of all statistical syntheses conducted. If meta-analysis was done, present for each the summary estimate and its precision (e.g. confidence/credible interval) and measures of statistical heterogeneity. If comparing groups, describe the direction of the effect.	√
	20c	Present results of all investigations of possible causes of heterogeneity among study results.	√
	20d	Present results of all sensitivity analyses conducted to assess the robustness of the synthesized results.	√
Reporting biases	21	Present assessments of risk of bias due to missing results (arising from reporting biases) for each synthesis assessed.	√
Certainty of evidence	22	Present assessments of certainty (or confidence) in the body of evidence for each outcome assessed.	√

PRISMA 2020 Checklist

Section and Topic	Item #	Checklist item	Location where item is reported
**DISCUSSION**			
Discussion	23a	Provide a general interpretation of the results in the context of other evidence.	√
	23b	Discuss any limitations of the evidence included in the review.	√
	23c	Discuss any limitations of the review processes used.	√
	23d	Discuss implications of the results for practice, policy, and future research.	√
**OTHER INFORMATION**			
Registration and protocol	24a	Provide registration information for the review, including register name and registration number, or state that the review was not registered.	√
	24b	Indicate where the review protocol can be accessed, or state that a protocol was not prepared.	√
	24c	Describe and explain any amendments to information provided at registration or in the protocol.	√
Support	25	Describe sources of financial or non-financial support for the review, and the role of the funders or sponsors in the review.	√
Competing interests	26	Declare any competing interests of review authors.	√
Availability of data, code and other materials	27	Report which of the following are publicly available and where they can be found: template data collection forms; data extracted from included studies; data used for all analyses; analytic code; any other materials used in the review.	√

From: Page MJ, McKenzie JE, Bossuyt PM, Boutron I, Hoffmann TC, Mulrow CD, et al. The PRISMA 2020 statement: an updated guideline for reporting systematic reviews. BMJ 2021;372:n71. doi: 10.1136/bmj.n71

For more information, visit: http://www.prisma-statement.org/
